# Phylogenomic Analysis of Wide‐Ranging Least Shrews Refines Conservation Priorities and Supports a Paradigm for Evolution of Biota Spanning Eastern North America and Mesoamerica

**DOI:** 10.1002/ece3.71263

**Published:** 2025-05-12

**Authors:** Tommy M. Galfano, Tommy M. Herrera, John B. Bulger, James N. Stuart, Jennifer K. Frey, Andrew G. Hope

**Affiliations:** ^1^ Division of Biology Kansas State University Manhattan Kansas USA; ^2^ Department of Biology Western University London Ontario Canada; ^3^ Department of Integrative Biology, Museum of Vertebrate Zoology University of California Berkeley California USA; ^4^ New Mexico Department of Game and Fish Santa Fe New Mexico USA; ^5^ Department of Fish, Wildlife, and Conservation Ecology New Mexico State University Las Cruces New Mexico USA

**Keywords:** conservation units, *Cryptotis parvus*, ddRADseq, mito‐nuclear discordance, peripheral endemism, population genomics

## Abstract

Anthropogenic global change is impacting the evolutionary potential of biodiversity in ways that have been difficult to predict. Distinct evolutionary units within species may respond differently to the same environmental trends, reflecting unique geography, ecology, adaptation, or drift. Least shrews (*Cryptotis parvus* group) have a widespread distribution across North America, yet systematic relationships and ongoing evolutionary processes remain unresolved. Westernmost peripheral populations have been prioritized for conservation, but little is known of their evolutionary histories or population trajectories. The broad range of this group of species is coincident with many other temperate taxa, presenting a hypothesis that diversification of least shrews follows a repeated process through the Pleistocene, leading to regionally diagnosable conservation units. We use genomic data and niche modeling to delimit species and conservation units of least shrews. Our results show that least shrews warrant recognition as multiple distinct species, along with geographically discrete infraspecific lineages of *C. parvus* (sensu stricto). Western peripheral populations are evolutionarily distinct based on nuclear, but not mitochondrial data, possibly reflecting mitochondrial capture during the last glacial phase. This population represents a relict conservation unit, consistent with both an “adaptive unit” and “management unit” based on non‐neutral and neutral divergence, respectively. Hindcast niche modeling supports growing evidence for a shared process of diversification among co‐distributed biota, and forecast modeling suggests continued future loss of suitable environmental niche in peripheral regions. Given mito‐nuclear discordance among samples of parapatric lineages, future environmental perturbation may continue to impact the genomic integrity of important conservation units, making ecological and genomic monitoring a critical need.

## Graphical abstract

Least shrews, distributed widely across North America and Mesoamerica, remain an enigmatic group in terms of their evolutionary history, biogeography, and ongoing responses to environmental change. Under a phylogenomic framework using reduced representation genome data, we investigated these dynamics across multiple scales of analysis. We found that the current taxonomy is in need of revision and provide updated nomenclature, systematic relationships, and conservation implications. This group provides a clear demonstration of diagnosable diversification across this vast study area.

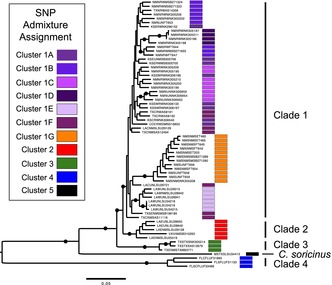

## 1 Introduction

Conservation and management efforts for wildlife have benefited in recent decades through the implementation of molecular methods (Avise 1989; Primmer 2009; Theissinger et al. 2023). Genetic analyses have greatly improved our understanding of evolutionary relationships among study taxa, and these analyses have either complemented or challenged traditional morphology‐based taxonomy and systematics (Coates et al. 2018; Dufresnes et al. 2023). Fundamentally, effective conservation requires a clear understanding of the relationships among study taxa and their ecological, geographic, and genomic attributes (Fenderson et al. 2020). However, even among mammals, true distributions, population trends through time, and the extent of evolutionarily distinct but morphologically cryptic diversity are often still poorly resolved (Herrera et al. 2022). Recent decades of phylogeographic studies, based often on organellar DNA and/or a few nuclear amplicons, have revolutionized the study of the spatial molecular ecology of nonmodel wildlife species, but the evolutionary histories of many species of conservation concern remain poorly understood (Hogg et al. 2022). Further, the constraints of only a few loci have often limited our ability to discern more complex evolutionary processes that might help to better define conservation priorities (Primmer 2009). For example, a single locus may remain unsorted among highly distinct taxa or may be discordant from true relationships. Particularly for infraspecific taxa, detecting neutral versus adaptive lineage‐specific divergence, coupled with accurate assessment of gene flow and genetic demographic trends, requires more substantial genomic coverage (Edwards et al. 2022). Here, we contribute research on a group of widespread but enigmatic small mammals based on a large genomic dataset that demonstrates many of the benefits of expanded genomic sampling, as well as multiple persistent constraints on our ability to define conservation priorities.

**FIGURE 1:**
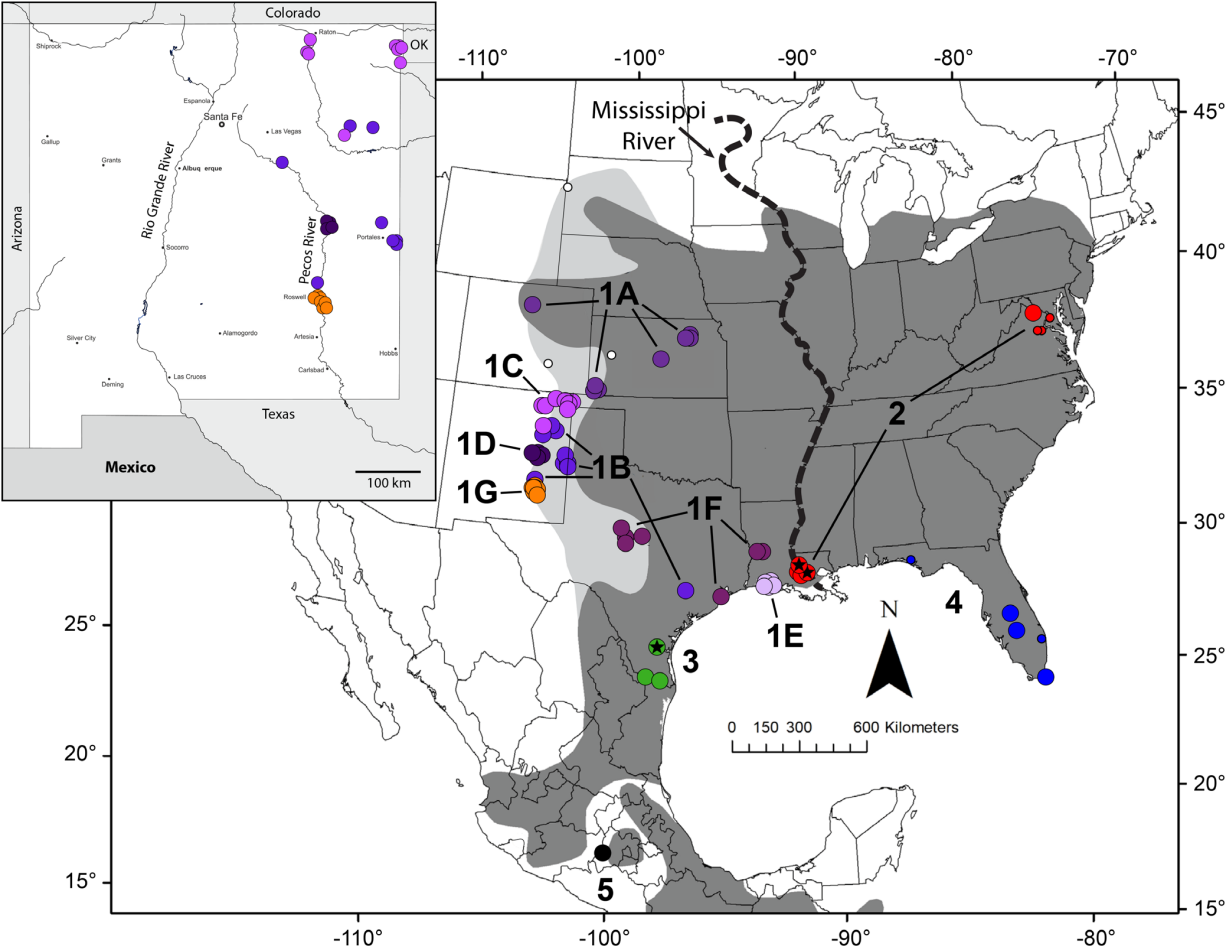
Map of the study area. Shaded regions represent the estimated range of *Cryptotis parvus*, based on Hall (1981; dark shading) and Barnes and Hoffman (2023; light shading), the latter suggestive of recent westward range expansion. Filled dots represent specimens sampled for genetic analyses and are colored and labeled according to genomic cluster assignments from the SNP admixture analysis (Figure 2). Within *C. parvus* , samples of *C. p. floridanus* within Florida are colored blue (cluster 4). Other species include *C. berlandieri* (cluster 3) from southern Texas colored dark green, and *C. soricinus* (cluster 5) from Mexico colored black. Small dots show specimen localities that were not included in genomic analyses, and are only colored if clade assignment was unambiguous from the mtDNA phylogeny. Localities with black stars show specimens that have a signal of mito‐nuclear discordance. Inset map provides additional spatial details for peripheral populations within New Mexico.

North American least shrews (the *Cryptotis parvus* group) have a broad distribution across North America, consisting of six nominal species, most with limited distributions through montane regions of Mexico (Woodman 2018). However, the recognized range of *Cryptotis parvus* (sensu stricto) is broad, from the Atlantic coast in the eastern U.S., north to roughly the Canadian/United States (U.S.) border, west to eastern portions of New Mexico, Colorado, and Wyoming, and south into eastern, central, and southern Mexico (Figure 1). Within the United States, there are two accepted subspecies: *C. p. floridanus*, occurring in Florida and northward through North Carolina, and *C. p. parvus*, which likely occurs in partial sympatry with *C. p. floridanus* in the southeast U.S. and through the remainder of the species' distribution. Other subspecies (*C. p. harlani*, *C. p. elasson*) have been discredited based on best available evidence (Hoffmeister 2002; Hutchinson 2010; Woodman 2018). A recent phylogenetic assessment of the genus *Cryptotis* noted evolutionary divergence between samples of *C. parvus* collected west and east of the Mississippi River (He et al. 2015), although sampling was limited. Hutchinson (2010) also performed a preliminary phylogeographic study of *C. parvus* in the United States based on short sequences from the mitochondrial genome (Cytb, CO1) and a single nuclear locus (Apolipoprotein B), where loci supported the distinction of three genetic lineages coincident with Florida, the remainder of the samples from east of the Mississippi River, and all samples west of the Mississippi River; a taxonomic revision of these shrews was suggested (Hutchinson 2010). Genomic analyses both within and among species of the *C. parvus* group have not been performed, and species limits among taxa remain unresolved.

Current conservation measures for *C. parvus* are based on incomplete information (Frey 2005). These shrews are among the smallest mammals and are locally rare, although they may be sampled in high densities in certain habitats or conditions (Whitaker 1974). Although traditional skin and skeleton specimens from early field sampling are represented in museum collections, relatively few robust genomic resources for this species exist for evolutionary analysis. The earliest records of locality of western peripheral populations of *C. parvus* (sensu stricto) were from as recent as 1961 in Eddy County, New Mexico (a population that is now considered extirpated due to extreme habitat degradation; New Mexico Department of Game and Fish [NMDGF] 2020). Subsequently, specimens were collected from multiple other localities in New Mexico (Hoditschek et al. 1985; Owen and Hamilton 1986; Shuster 1989; Hafner and Shuster 1996; Frey 2005; NMDGF 2020). Given a lack of knowledge of the distributional extent, population status, or habitat requirements of least shrews in New Mexico, *C. parvus* was listed as threatened by the New Mexico Department of Game and Fish in 1985 and is a Species of Greatest Conservation Need (SGCN) under the New Mexico State Wildlife Action Plan (Jones and Schmitt 1997; NMDGF 2020, 2024).

All western peripheral populations of *C. parvus* are currently managed as a single taxon. However, early study of western peripheral populations based on genetic allozyme data coupled with morphometric analysis of cranial measurements identified two putatively distinct lineages of shrews in New Mexico, suggesting that a population associated with arid‐land spring‐fed wetlands of the Roswell Artesian Basin that includes Bitter Lake National Wildlife Refuge (hereafter Chaves County; Land and Huff 2010) was a distinct relict of previously more widespread populations of *Cryptotis berlandieri* (Hafner and Shuster 1996). Our research here is therefore a result of ongoing efforts to improve understanding of the conservation status of this shrew towards recovery plan efforts that will ensure long‐term persistence of robust, representative, and secure populations of least shrew in New Mexico, such that they no longer require protection (NMDGF 2020). Given that *Cryptotis parvus* is listed as an SGCN in New Mexico, coupled with preceding research that suggests current taxonomy from these western populations may not adequately reflect the existing diversity, a number of important conservation questions are relevant for investigation: Are these peripheral shrews a single evolutionarily significant unit? Which other populations are most closely related to these peripheral populations, and how closely? Are these populations new, resulting from recent range expansion, or post‐Pleistocene relictual endemics, and what are their continued demographic trends?

Ecologically, *C. parvus* is seemingly a generalist in its habitat associations, occurring within pine forests and swamps in the southeastern U.S., eastern deciduous forests, prairie grasslands of the Great Plains, and associated with isolated and ecologically unique wetlands in southeast New Mexico (Whitaker 1974). Given a lack of adherence to specific habitat types, the combined range of the *C. parvus* group is generally coincident with mesic‐temperate habitats of eastern North America and Mesoamerica, and distributions may ultimately be defined either by climatic constraints, or, in some populations, may be strongly tied to local habitats that enable persistence in regions outside of climatic niche space. Either way, the combined distributional envelope of the *C. parvus* group is shared by biota that span diverse floral and faunal assemblages. A paradigm for generalizable processes of diversification of these co‐distributed taxa is emerging (e.g., Martin and Harrell 1957; Stull 2023), with a mechanism for evolution through time consistent with cyclic responses to Pleistocene environmental change (Stull 2023). Genomic methods, coupled with distributional modeling, allow for testing of these comparative hypotheses of shared evolution across disparate groups of species, providing predictive power for delineating genetically distinct regional communities. Diagnosis of both distributional *patterns* of regional evolutionary hotspots and the *processes* that developed these patterns through time would provide critical resources for strengthening large‐scale regional conservation plans. We therefore extend our phylogenomic analyses of the *C. parvus* group to assess adherence of these small mammals to a shared biotic history of differentiation.

This study represents multiple priorities toward improving conservation practices. The first is to apply modern genomic methods and analyses to refine our knowledge of distinct units of biodiversity within the *C. parvus* group, both between and within species. The second is to perform population genomic analyses for *C. parvus* (sensu stricto) with a focus on peripheral populations to guide local management of potentially imperiled populations, but including a range‐wide assessment of diversity to emphasize support for regional findings from a broader context. The third is to leverage knowledge of the evolutionary history of least shrews to contribute to our understanding of comparative evolution among species that are co‐distributed through eastern North America and Mesoamerica through discussion of studies on other taxa in the context of our results, including modeled predictions for distributional change through time. Effective conservation will likely hinge on multiple scales of analytical resolution. Ultimately, we provide a demonstration of how phylogenomic analysis can effectively advance conservation management practices at large, while revealing remaining hurdles in how we describe and document biodiversity.

## 2 Methods

### 2.1 Sampling and DNA Extraction

Samples for this project were acquired from a combination of tissue loans from natural history collections, as well as inclusion of published Cytb data downloaded from GenBank (Appendix S1). Sampling included localities from throughout the range of the *Cryptotis parvus* group, but we focused greater sampling on western peripheral populations of conservation concern (Figure 1). Samples include representation of most species belonging to the *C. parvus* group, including *C. parvus* (sensu stricto; *n* = 83), *C. berlandieri* (*n* = 3), *C. orophilus* (*n* = 1; Cytb only), *C. soricinus* (*n* = 1), and *C. tropicalis* (*n* = 1; Cytb only). No samples of *C. pueblensis* (also within this group) were available for genetic analysis. Additional outgroups included *C. goldmani* (*n* = 1), *Blarina carolinensis* (*n* = 2), *B. brevicauda* (*n* = 1), and *Notiosorex crawfordi* (*n* = 2). None of the additional outgroups were sequenced across both Cytb and nuclear loci. Within *C. parvus* (sensu stricto), we performed analyses considering four separate groups, including *C. p. floridanus*, all samples of *C. p. parvus* east of the Mississippi River (hereafter East), and all samples of *C. p. parvus* west of the Mississippi River (hereafter West). Additionally, samples from the West group included western peripheral populations of particular interest for conservation efforts by the State of New Mexico from Chaves County, NM, so we included statistics based on these samples separately. DNA was extracted from either muscle or liver tissue for each sample using the Qiagen DNeasy Blood and Tissue kit and accompanying protocol (Qiagen, Valencia, California).

### 2.2 DNA Sequencing

The full mitochondrial Cytb gene (1140 bp) was amplified for each sample using primers MSB05 and MSB014 developed previously (Hope et al. 2010). Polymerase chain reaction conditions were: 6 min at 94.0°C, 40 cycles of 94.0°C for 25 s, 48.0°C for 30 s, and 72.0°C for 1 min, followed by 72.0°C for 15 min, then held at 10.0°C. PCR products were cleaned using the ExoSAP‐it protocol (Bell 2008). Sanger sequencing was performed by Genewiz LLC on an ABI 3730 (South Plainfield, NJ). Postsequencing, raw reads were cleaned and aligned using Geneious Prime (Kearse et al. 2012). Final sequences were trimmed to 1100 bp (range 900–1100 bp).

Nuclear ddRADseq sequencing and variant calling closely followed methods outlined in Combe et al. (2022) and Hope et al. (2023). Genomic DNA was quantified using Quant‐iT Picogreen dsDNA Assay (Invitrogen). Samples with good yields (> 100 ng) of high molecular weight DNA were submitted to the University of Minnesota Genomics Center (Minneapolis) for double digest restriction site associated DNA sequencing (ddRADseq; Peterson et al. 2012), using *Sbf*I and *Taq*I restriction enzymes (New England Biolabs). Libraries were sequenced with 150 bp single‐end reads on a NextSeq 550 (Illumina, USA), using a High‐Output flow cell.

### 2.3 SNP Identification and Filtering

Raw reads were trimmed and padding sequences were removed with gbstrim.pl. (https://bitbucket.org/jgarbe/gbstrim/src/master/gbstrim.pl). Trimmed reads were filtered for quality with *process_radtags* in Stacks v2.54 (Rochette et al. 2019). Briefly, this removes reads with uncalled bases and low‐quality scores using a sliding‐window approach. We aligned reads from each individual to a *C. parvus* reference genome assembly (Ovir.te_1.0; GenBank assembly accession: GCA_021461705.1) using the BWA‐MEM algorithm of the Burrows‐Wheeler Alignment tool v0.7.17 (Li 2013), with default settings. Mapped reads were sorted and indexed using SAMtools (Li 2011). Loci were discovered and SNPs called with the *ref_map.pl* pipeline in Stacks, using M = 3, representing a minimum coverage option, which optimized the number of polymorphic loci while minimizing the number of potentially paralogous loci (Rochette and Catchen 2017). We reduced SNPs to one per locus (‐write‐single‐snp) using the *populations* module in Stacks and filtered for quality and completeness using R v4.1.2 (R Development Core Team 2018), packages *SNPfiltR* (DeRaad 2022) *sambaR* (de Jong et al. 2021) and *vcfR* (Knaus and Grünwald 2017). We set a minimum read depth of 5 and genotype quality of 30, filtered heterozygotes for an allele balance ratio between 0.25–0.75, set a maximum read depth of 50, a maximum proportion of missing data per sample of 0.58, a maximum proportion of missing data per SNP of 0.8, and loci must have been present in 75% of individuals. These values were determined in *SNPfiltR* by iteratively assigning cut‐offs for missing data, checking PCAs to make sure missing data were not driving spatial differentiation, and assigning final values that resulted in most data coupled with robust inference. This SNP set was used for all subsequent analyses. A number of recent studies have demonstrated the utility of ddRADseq data for multi‐species phylogenetic analyses, although with precautions for careful assessment of SNP yield versus missing data (e.g., Leaché et al. 2015; Leache et al. 2015; Salas‐Lizana and Oono 2018). Given that there was a large amount of missing data for *C. floridanus* , *C. soricinus* , and *C. berlandieri* in the SNPs used for the analysis of the *C. parvus* ingroup, we used relaxed filters for RAxML analysis of multiple species (Table S1). For analyses using the outgroup SNP set, we implemented the same coverage, genotype quality, and allele balance ratio filters, but kept every individual and set the maximum proportion of missing data per SNP at 0.6. Finally, loci were tested for deviation from Hardy–Weinberg Equilibrium using the R package *pegas* (Guo and Thompson 1992; Paradis 2010) with the exact test and 100 Monte Carlo permutations. These filters retained 22,786 SNPs with 2.87% missing data overall (Table S1).

### 2.4 Detection of Loci Under Selection

To identify and designate different conservation units in a population as outlined in Funk et al. (2012) and implemented by Barbosa et al. (2018), SNP data were analyzed depending on their relative neutrality. Whole genome divergence (considering all genetic loci examined regardless of neutrality) of lineages is considered to constitute ESUs for traditional conservation of discrete genetic diversity (Moritz 1994). However, more recently, consideration of adaptive potential has resulted in recognition of additional management‐based units of analysis. The first are Adaptive Units (AUs) that identify divergent populations reflected by non‐neutral outlier loci, suggesting that such units are geographically unique due to derived local adaptations through natural selection in response to divergent environmental pressures (Funk et al. 2012). Conversely, Management Units (MUs) from a genetic perspective consider geographically discrete populations that have experienced neutral evolutionary divergence from each other, most often due to extended allopatry, accompanied by genetic drift, and should be considered independently for ongoing management (Funk et al. 2012).

Subsequent clustering analyses were therefore repeated using all genomic loci, only neutral loci, and only non‐neutral (potentially adaptive) loci. Separation of the full dataset into non‐neutral and neutral datasets was performed using two methods and including all samples. We first used BayeScan v.2.1 (Foll and Gaggiotti 2008). Briefly, prior odds of 10 (prior belief that a selection model is 1/10 as likely as a neutral model for a given SNP), 100, and 1000 were used for identifying the top candidates of the selected loci and a total of 50,000 reversible‐jump Markov Chain Monte Carlo iterations were run with a thinning interval of 10, following 20 pilot runs of 5000 iterations each and a burn‐in length of 50,000. Second, we used *PCadapt* v4.1.0 (Luu et al. 2017), a multivariate method that ascertains population structure using principal component analysis (PCA) and then identifies markers under putative selection as those excessively correlated with population structure. A scree plot of the first 20 principal components (termed *K* in *PCadapt*) indicated that the optimal *K* from our data was 5 for computing correlations between loci and *K* principal components. We used Benjamini and Hochberg's (1995) method for correction of the false discovery rate in both BayeScan and *PCadapt* at *α* = 0.05. Based on the results of both analyses, we separated our ddRAD‐seq data into neutral loci and non‐neutral loci, using a custom script (https://github.com/fraser‐combe). The resulting VCF files included 21,451 neutral loci and 1,335 non‐neutral loci.

### 2.5 Clustering Analyses of Population Structure

We used two approaches for clustering analyses. The first approach used PopCluster v 1.4.0.0 (Wang 2022), based on the full SNP dataset. We tested for a range of K values, from two to 20, and ran 10 iterations for each K value, determining the best value of K using the DLK2 estimator. The DLK2 estimator is similar to the Evanno method but does not require the mean and standard deviation values of log‐likelihood values between K runs because of the convergence of the clustering analysis caused by a simulated annealing algorithm (Wang 2022). Clustering plots were visualized using the PopCluster GUI (Wang 2022).

The second approach used principal components analysis (PCA) performed in the R package *dartR* (Gruber et al. 2018). The number of principal components retained was determined by a scree plot where the optimal number of principal components was identified by retaining the first value on the plot where the slope of the line drastically flattens. This analysis was repeated three times using the full SNP dataset, neutral, and non‐neutral locus sets. In addition, for each dataset, the analysis was performed once including all taxa, and again including only samples of *C. p. parvus* (East, West, Chaves County groups). Given small samples for some taxa, analyses including all available samples were not able to provide confidence ellipses to assess overlap between groups. Re‐running analyses with only *C. p. parvus* samples allowed for more fine‐scale population analysis within this group.

### 2.6 Phylogenetic Analyses

We estimated two independent genealogies for the Cytb locus, including incomplete sequences and without assigning haplotypes. The first included all available sequences of all represented species, and the second included only sequences of *C. p. parvus*. We produced Bayesian chronograms in BEAST2 v2.6.3 (Bouckaert et al. 2019), setting all parameters in BEAUti, part of the BEAST2 software package, and estimating the substitution model through the use of the bModelTest package, also in BEAST2 (Bouckaert and Drummond 2017). We applied a relaxed clock: uncorrelated log‐normal molecular clock model and set the mutation rate to 0.055 (5.5% per million years; Hope et al. 2010). We used empirical base frequencies and a constant population size tree prior, with other parameters run with default settings. We ran MCMC for 50 million generations, sampling every 1000 generations, with the first 1000 trees discarded as burn‐in. Stationarity of MCMC runs was assessed in Tracer v1.7 (Rambaut et al. 2018). We annotated tree files in TreeAnnotator (BEAST2 package). Chronograms were visualized with posterior probabilities in FigTree v1.3.1 (Rambaut 2009) and reported as a mid‐point rooted tree.

Phylogenies based on SNP data were performed using a maximum likelihood (ML) approach in RAxML v8.2.12 (Stamatakis 2014) and were repeated for the full SNP dataset, and for non‐neutral and neutral locus sets. In accordance with recommendations in Leaché et al. (2015), an ascertainment bias was applied to the likelihood calculation to correct for the lack of invariant sites in an SNP‐based phylogeny. The SNP dataset was converted from VCF to phylip format. ML analysis was run using 100 bootstrap replicates, the GTR + Gamma substitution model, and the Lewis method for ascertainment bias correction (Lewis 2001).

### 2.7 Diversity and Demographic Analyses

We assessed genetic diversity, pairwise divergence (for Cytb), and demographic trends for diagnosable evolutionary lineages based on clades recovered from phylogeny reconstructions. Complicating these efforts due to recovered mito‐nuclear discordance, support for lineages and sample inclusion within lineages varied depending on whether we considered the Cytb phylogeny or the SNP phylogeny. However, major lineages were generally consistent across both datasets and included samples of *C. berlandieri* , the single sample of *C. soricinus* , samples of *C. p. floridanus*, all samples of *C. p. parvus* East excluding Chaves County specimens, which were analyzed separately, and all samples of *C. p. parvus* West.

For the Cytb dataset, we used DnaSP v6.12 (Rozas et al. 2017) to calculate genetic diversity statistics for each lineage, including pairwise sequence divergence, number of haplotypes (h), number of segregating sites (S), haplotype diversity (Hd), nucleotide diversity (π), Tajima's *D* (Tajima 1989), and Fu's F‐statistic (Fu 1997), the latter two to test for demographic changes in effective population size (*N*_e_) but were only applied to those populations of sufficient sample sizes, including *C. p. floridanus*, West, and Chaves County.

For SNPs, we used the full SNP dataset within the Stacks pipeline, *populations* module to calculate π and the inbreeding coefficient (*F*_IS_). In addition, estimates of observed heterozygosity (*H*_O_) and expected heterozygosity (*H*_E_) were obtained using the R packages hierfstat (Goudet 2005) and adegenet (Jombart 2008), respectively. Pairwise *F*_ST_ values for each lineage were also generated in R using the packages hierfstat and mmod (Winter 2012). Finally, for West and Chaves County populations, we examined temporal changes in N_e_ using Stairway Plot v2.1 (Liu and Fu 2020). This approach uses a folded SNP frequency spectrum (SFS), which does not have to take ancestral allelic conditions into consideration and does not assume selectively neutral markers. To generate the SNP frequency spectrum, sorted BAM outputs (full locus sequence data) from the Stacks *process_radtags* module were aligned to the *Cryptotis parvus* reference genome. In the SAMtools module, using ANGSD v0.940 (Korneliussen et al. 2014), maximum likelihood estimates were generated for each population subset. All reads were subjected to a set of quality filters: Presence in 80% or more of the members of the populations has a mapping quality score of at least 25 and a base quality of at least 13. This produced 1,731,131 sites for Chaves County and 1,833,177 sites for West samples. We assigned a generation time of one year and a mutation rate of 1.2 × 10^−8^ per site per generation (Liu et al. 2014). We reported median population sizes with 95% confidence intervals calculated based on 200 bootstrap replicates.

### 2.8 Ecological Niche Modeling Predictions

To obtain species occurrence data in R v4.1.2, we used the package *rgbif* v3.6.0 (Chamberlain et al. 2022). *Cryptotis parvus* is among the most difficult mammal species to identify (Kays et al. 2022), and genetic analyses presented here clearly show the presence of cryptic diversity within the *C. parvus* group, so the inclusion of data points that represent only *C. parvus* (sensu stricto) presented difficulties. First, GBIF spatial outlier points were removed to reduce the likelihood of the inclusion of misidentified records. Spatial outlier points were defined by their proximity to data points falling within the International Union for Conservation of Nature (IUCN) Red List range of *C. parvus* as well as to genetically verified data points to further account for distributional underestimation from the IUCN Red List (Hughes et al. 2021). We included data points north of and coincident with the panhandle of Florida so as to exclude *C. p. floridanus*. We included data points falling within the new estimated western range limit of *C. parvus* (sensu stricto) as summarized by Barnes and Hoffman (2023). However, based on all other analyses presented here, and as discussed further below, all samples of putative *C. parvus* from south of the Rio Grande along the U.S. Mexico border appear to represent *C. berlandieri* or other species of the *C. parvus* group, as also supported by the most current taxonomic designations and associated distributions (Woodman 2018). As such, specimen records south of the U.S. border were excluded. The maximum distance between neighboring points that were genetically verified and points that were within the modified IUCN Red List range map was used as a threshold to identify spatial outlier points. Any points falling outside of this buffer were excluded. Following data cleaning, the *spThin* package v0.2.0 (Aiello‐Lammens et al. 2015) was used to spatially thin the dataset to reduce sampling bias. The “thin” parameter was calculated with the Average Nearest Neighbor Index (Evans and Ram 2021), producing the expected mean distance between points assuming a random distribution.

The bioclimatic variables used in niche models were downloaded from WorldClim v2.1 (Fick and Hijmans 2017) at 2.5′ spatial resolution. All 19 variables were included for modeling regardless of variable correlation as the Maxent algorithm is robust against collinearity (Feng et al. 2019). The bioclimatic variables were cropped to a study area defined by a 150 km buffer around occurrence points to represent spatial availability and use (Soberón 2010). We built and evaluated ENMs using the ENMeval package v2.0.3 (Kass et al. 2021), using the Maxent algorithm (Phillips et al. 2017). We used the “block” method to partition occurrences into training and testing datasets and applied clamping to model training to avoid the inflation of estimated suitability when projecting to conditions outside of the study area (Merow et al. 2013). Feature class inputs used for model evaluation included linear, quadratic, product, threshold, and hinge, along with all possible combinations of these five classes. We tested regularization multipliers 1:10. We validated the models with a significance test of Partial ROC using the *kuenm* package v. 1.0 (Cobos et al. 2019). We then selected models based on the Akaike Information Criteria (AIC) to maximize model simplicity and provide increased discrimination between models (Escobar et al. 2018). Of the models providing AICc values < 2, we selected the final model based on the lowest 10% omission rate (Anderson et al. 2003). The final ENM was projected across contiguous North America using a logistic output format. We used pseudoabsence data and the *modEvA* package v3.0 (Liu et al. 2005) to calculate a threshold for suitability based on the “prevalence” output.

To build hindcast models projected to the Last Glacial Maximum, we used Global Circulation Models (GCMs) from WorldClim v1.4, including CCSM4, MIROC‐ESM, and MPI‐ESM‐P. We applied the same suitability threshold calculated from modern climate conditions with *modEvA*. We then combined results from each GCM projection to define areas of historic suitability as regions where at least two of the three GCMs agreed. The same approach was used for forecasting niche models projected to 2041–2060 and 2061–2080 climatic conditions. We used three GCMs, including BCC‐CSM2‐MR, CNRM‐CM6‐1, and CanESM5, and defined areas of suitability where at least two GCM projections agreed on suitable niche space. We used Socioeconomic Pathway 2‐4.5, which represents a moderate‐emission scenario assuming current socioeconomic and climatic trends without major policy shifts toward lower carbon emissions.

## 3 Results

### 3.1 Sampling and Geographic Distribution of Genetic Diversity

**FIGURE 2:**
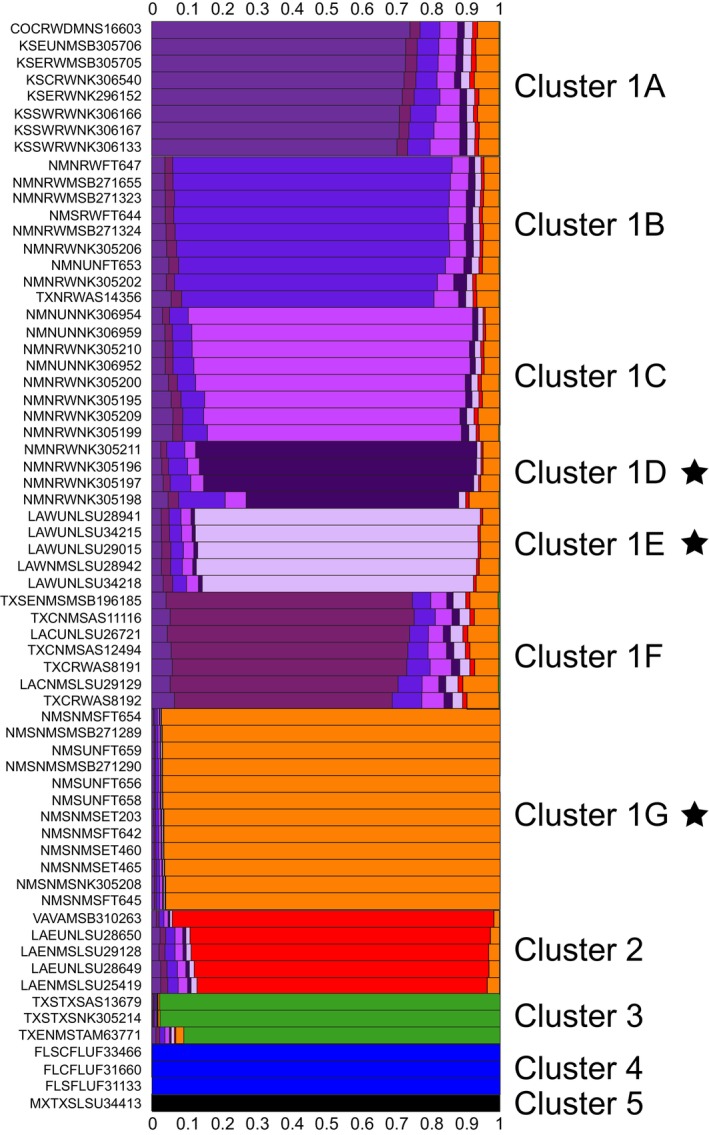
Admixture plot showing distinct genomic clusters of individuals of *Cryptotis* based on the best supported value of *K* = 11, and assignment of SNP loci to each cluster. Clusters 1A–1G illustrate genomic differentiation within *C. parvus parvus* West, with Cluster 1G representing shrews from Chaves County, New Mexico. Cluster 2 = *C. p. parvus* East shrews; Cluster 3 = *C. berlandieri* ; Cluster 4 = *C. p. floridanus*; Cluster 5 = *C. soricinus* . Sample labels correspond to specimen data provided in the Appendix S1. Stars next to cluster names show groups of samples that are also recovered as monophyletic and well supported through ML phylogeny estimation based on all loci, and only neutrally evolving loci, suggesting Evolutionary significant units and management units respectively. Cluster 1G was in addition recovered as a well‐supported monophyletic group based on outlier loci, suggesting 1G is also a significant adaptive unit.

Based on our sampling from across the distribution of the *C. parvus* group, genomic diversity is structured geographically (Figure 1), where clusters from the admixture analysis (Figure 2) generally consist of samples in close proximity to each other, in relation to samples from other clusters. We use these cluster assignments (Figure 2; 1A–1G, 2, 3, 4, 5) throughout the results section to relate groupings from different analyses.

### 3.2 Clustering Analyses of Population Structure

**FIGURE 3:**
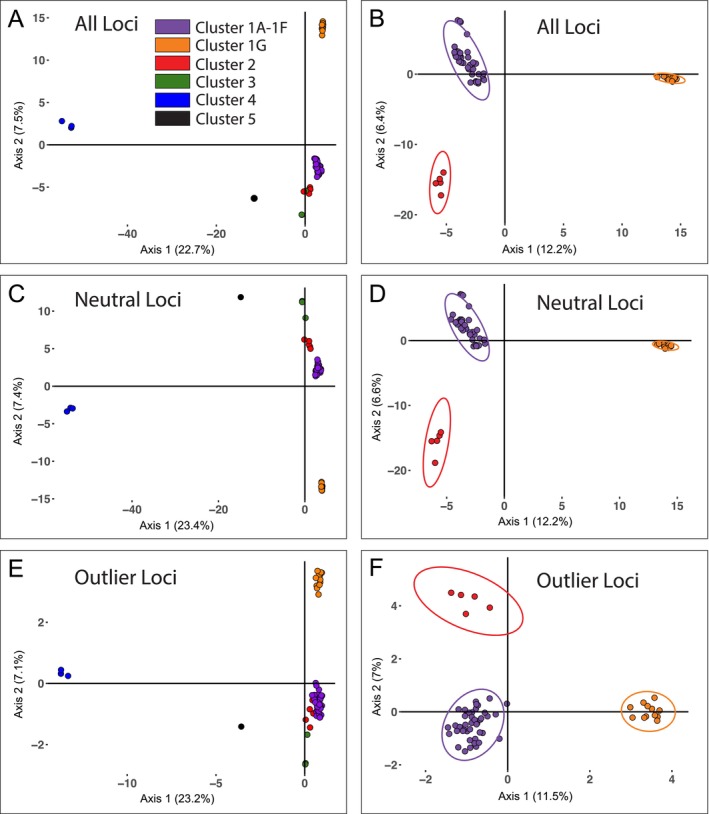
DAPC plots considering, (A, B) all loci (22,786 SNPs); (C, D) putatively neutral loci (21,451 SNPs); (E, F) outlier locrocidurai (1335 SNPs) and inclusive of all taxa with available nuclear data that are colored and labeled according to clusters recovered in the Admixture analysis (Figure 2). Plots on the left are inclusive of all samples available, whereas plots on the right include only samples of *C. parvus* (*sensu stricto*), excluding *C. p. floridanus*. Clusters 1A–1G illustrate genomic differentiation within *C. parvus parvus* West, with Cluster 1G representing shrews from Chaves County, New Mexico. Cluster 2 = *C. p. parvus* East shrews; Cluster 3 = *C. berlandieri* ; Cluster 4 = *C. p. floridanus*; Cluster 5 = *C. soricinus* . All plots illustrate clusters based on the first two principal component axes. Ellipses are excluded as some clusters have ≤ 3 individuals.

Based on Admixture analysis, the best supported value for the number of clusters was *K* = 11 (Figure S1). This included seven clusters (Figure 2; 1A–1G) within *C. p. parvus* west of the Mississippi River with varying degrees of unsorted (or admixed) loci between clusters. The most well‐defined cluster of these western populations was the sample from Chaves County (Figure 2; 1G; Figure S1). Samples east of the Mississippi River formed a separate cluster, although with greater similarity to clusters 1A–1F than to 1G. A separate cluster included three samples of *C. berlandieri* , with a single sample suggestive of potential admixture with *C. p. parvus* West, while the other two samples are highly distinct. A cluster containing *C. p. floridanus* is also highly distinct, as is the final cluster of *C. soricinus* . Clusters recovered from admixture analysis were also generally recovered from PCA plots, although with much less distinct differentiation among samples from *C. p. parvus* west of the Mississippi River (Figure 3). The exception to this is the Chaves County samples, which are again highly distinct considering all locus sets (Figure 3).

### 3.3 Phylogenetic Analyses

**FIGURE 4:**
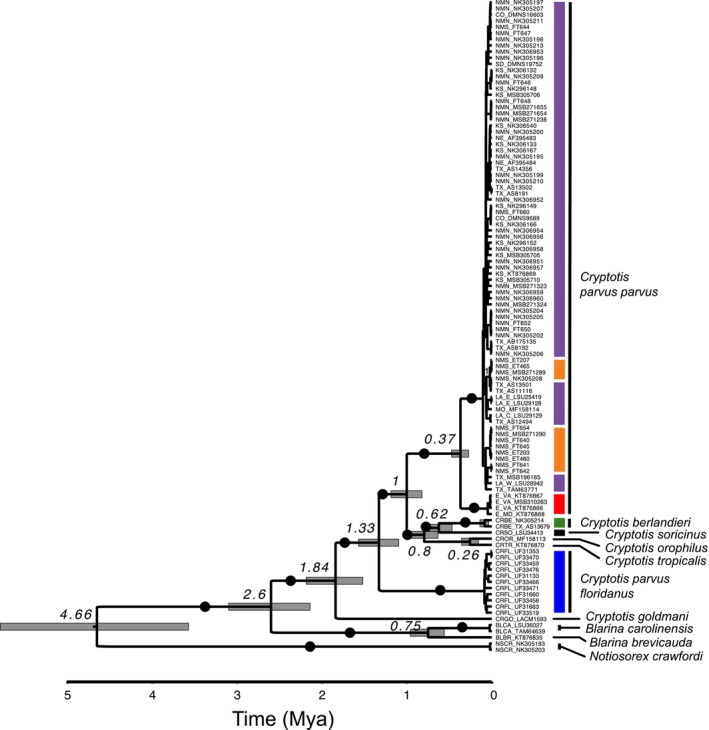
Bayesian mtDNA cytochrome b chronogram as estimated in BEAST for all *Cryptotis parvus* samples sequenced and including multiple outgroup taxa. Node values represent estimated coalescence dates and bars represent associated 95% confidence interval of coalescence estimates. Black dots show ≥ 0.95 posterior probability estimates at the proceeding node. The scale bar shows tree depth in millions of years before present based on an estimated mutation rate of 5.5% per million years. Colors reflect cluster assignment based on nuclear SNP loci as reported in Figure 2. Orange bars reflect a lack of monophyly of Chaves County specimens based on mitochondrial data.

The Cytb phylogeny supports multiple shrew lineages coincident with regional geographic distributions (Figure 4). The deepest divergence among taxa associated with the *C. parvus* group is between samples representing *C. p. floridanus* and all other taxa dated to 1.33 Ma (95% CI = 0.88–2.11 Ma). This result renders *C. parvus* paraphyletic with respect to a clade containing all other species of the *C. parvus* group sampled (where *C. p. parvus* is more distantly related to *C. p. floridanus* than to *C. berlandieri* , *C. soricinus* , *C. orophilus*, and *C. tropicalis* ). This group of species coalesces with each other at 0.8 Ma (CI = 0.46–1.09 Ma) and shares a common ancestor with *C. p. parvus* at 1 Ma. Divergence of *C. soricinus* from samples of *C. berlandieri* was dated at 0.62 Ma (CI = 0.28–0.84 MA). Divergence between *C. tropicalis* and *C. orophilus* was dated to 0.26 Ma (CI = 0.1–0.47 Ma). Of interest, divergence between West and East clades of *C. p. parvus* is well supported as reciprocally monophyletic and is deeper (0.37 Ma; CI = 0.22–0.68 Ma) than divergence between *C. tropicalis* and *C. orophilus*. Within the West clade of *C. p. parvus*, there are multiple well‐supported subclades although with no discernible relationship to geographic regions (Figure 4; Figure S2). There is no supported distinction of Chaves County shrews as unique from the remainder of the West clade from mitochondrial data.

**FIGURE 5:**
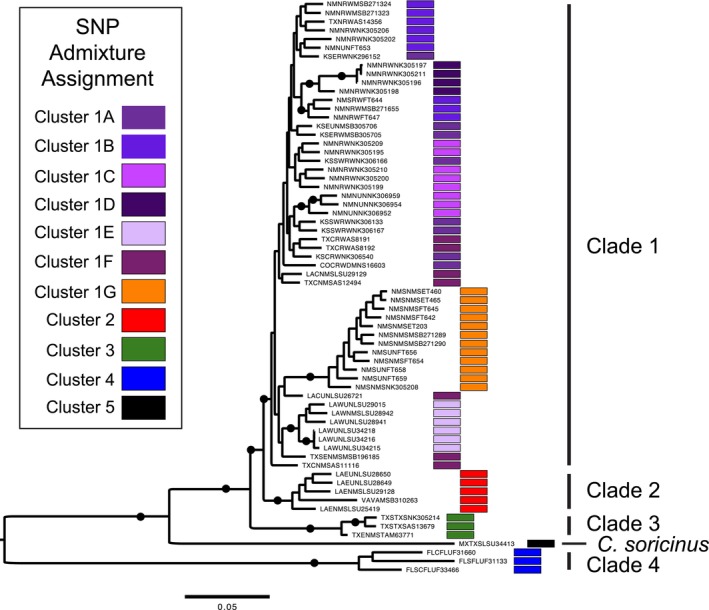
RAxML phylogeny for all samples with available nuclear data, based on all loci (22,786 SNPs). Black dots show ≥ 0.95 bootstrap support at the proceeding node. Bars next to terminal branches are colored according to cluster assignment based on nuclear SNP loci as reported in Figure 2.

Based on all SNP data, the RAxML phylogeny recovered the same well‐supported lineage relationships among species of the *C. parvus* group as shown for mitochondrial data (Figure 5). Again, the clade representing *C. p. floridanus* is the most highly divergent of all *C. parvus* group species, rendering *C. parvus* (sensu stricto) paraphyletic. These relationships are also supported by only neutral loci (Figure S3), although for outlier loci, *C. berlandieri* is grouped within *C. p. parvus* (Figure S4). Notably, based on all locus sets, *C. p. floridanus*, *C. soricinus* , and *C. berlandieri* are highly distinct from the remainder of *C. p. parvus*. Within *C. p. parvus*, all specimens east of the Mississippi river form a monophyletic group across all locus sets, as do all specimens from Chaves County, New Mexico, except for a single specimen (FT644; Table S2). Further, a group of specimens from southeast Louisiana and a group of specimens from Bosque Redondo, New Mexico, also each form monophyletic groups based on all loci and neutral loci but not non‐neutral loci. The RAxML phylogenies also exposed several specimens with mito‐nuclear discordance, including a single specimen of *C. berlandieri* with a mitotype of *C. p. parvus* and two specimens from the East clade of *C. p. parvus* collected in Baton Rouge, LA (east of the Mississippi) that also have mitotypes of *C. p. parvus* from the West clade. Finally, although there is no resolved lineage associated with Chaves County based on mitochondrial data, these specimens are clearly sorted and highly distinct based on nuclear data.

### 3.4 Diversity and Demographic Analyses

Cytb sequences provided comparative data for depth of lineage divergence among *Cryptotis* shrews relative to divergences based on a wealth of other mammalian phylogeographic studies. Within the broader *C. parvus* group of shrew species, samples representing *C. p. floridanus* were ~10% divergent from the remainder of *C. parvus* as well as from *C. berlandieri* , *C. soricinus* , *C. orophilus*, and *C. tropicalis* (Table 1). Divergence between *C. berlandieri* and the clade containing *C. orophilus* and *C. tropicalis* was ~7.5%. Divergence between *C. orophilus* and *C. tropicalis* , at ~2.9%, and divergence between West and East clades of *C. p. parvus* were ~3%. Nucleotide diversity from Cytb was relatively high within the *C. p. parvus* West group, although most of the diversity was within individuals from Chaves County, and diversity across the remainder of the distribution was low (Table 2). Highly significant expansion statistics for specimens in clusters A–F (excluding cluster G from Chaves County) also suggest demographic expansion, which is congruent with an observed extension of the range of this species along the western periphery (Barnes and Hoffman 2023). However, Chaves County specimens showed a strongly positive value for Tajima's D, supporting a signal of population persistence coupled with demographic contraction.

**TABLE 1:** Genetic differentiation statistics between major groups within *Cryptotis parvus*, and between nominal species within *Cryptotis*.

*F*_ST_\% Div.	*C. parv* (All)	Clust A–F	Clust G	East	*C. berl*	*C. flor*	*C. sori*	*C. trop*	*C. orop*
*C. p. parvus* (All)	—	—	—	—	9.407	10.912	8.082	8.338	9.482
*C. p*. (Clust A–F)	—	—	0.765	2.782	9.116	11.609	8.314	—	—
*C. p*. (Clust G)	—	0.047	—	3.296	9.087	10.926	7.947	—	—
*C. p*. (East)	—	0.046	0.183	—	9.229	10.702	8.759	—	—
*C. berlandieri*	—	0.072	0.261	0.183	—	9.911	5.998	7.525	7.343
*C. p. floridanus*	—	0.169	0.350	0.312	0.394	—	9.464	9.637	10.155
*C. soricinus*	—	0.134	0.403	0.362	0.559	0.438	—	7.814	6.975
*C. tropicalis*	—	—	—	—	—	—	—	—	2.927
*C. orophilus*	—	—	—	—	—	—	—	—	—

*Note:* Numbers above the diagonal are average percentage divergence between taxon pairs based on mtDNA cytochrome b data and numbers below the diagonal are *F*_ST_ values based on 22,786 nuclear SNP loci.

**TABLE 2:** Diversity statistics for 1100 bp of the mtDNA cytochrome *b* gene for *Cryptotis* taxa. Those taxa with only a single sequence were excluded from analysis.

	*N*	*h*	*S*	Hd	π	T's *D*	Fu's Fs
*C. p. parvus* (All)	84	21	40	0.694	0.0057	—	—
*C. p*. (Clust A–F)	68	22	30	0.770	0.0021	−2.181***	−16.00***
*C. p*. (Clust G)	12	5	16	0.788	0.0079	1.374	3.311
*C. p*. (East)	4	2	3	0.500	0.0021	—	—
*C. berlandieri*	2	2	11	1.000	0.0100	—	—
*C. p. floridanus*	11	10	23	0.982	0.0053	−1.417	−4.211*

*Note:* Significance of tests of demographic expansion are indicated by asterisks where *significant at *p* < 0.05, and ***significant at *p* < 0.001.

Abbreviations: π, nucleotide diversity; Fu's Fs, Fu's Fs statistic; h, number of haplotypes; Hd, haplotype diversity; *N*, sample size; *S*, number of segregating sites; T'SD, Tajima's *D* statistic.

Based on SNP data, values of observed heterozygosity were generally consistent with expected heterozygosity, and coupled with low values of F_IS_, suggest that there are no strong signals of inbreeding within lineages (Table 3). Nucleotide diversity was generally high within *C. p. floridanus*, and East and West lineages of *C. p. parvus* (the latter not including Chaves County), but lower within Chaves County (Table 3). Within *C. parvus* (sensu stricto), *C. p. floridanus* was most highly differentiated from other lineages (Table 1). The West lineage of *C. p. parvus* (not including Chaves County) was roughly equally differentiated from both Chaves County and the East lineage, but Chaves County exhibited much higher differentiation from the East lineage reflecting a longitudinal gradient of differentiation across these three nuclear lineages.

**TABLE 3:** Diversity statistics for nuclear SNPs for *Cryptotis* taxa.

	*N*	Private	*H*_O_	*H*_E_	π	*F*_IS_
*C. p*. (Clust A–F)	39	5731	0.093	0.122	0.124	0.132
*C. p*. (Clust G)	12	1043	0.070	0.079	0.083	0.039
*C. p*. (East)	5	1493	0.091	0.103	0.116	0.055
*C. berlandieri*	3	851	0.061	0.057	0.068	0.012
*C. p. floridanus*	3	2577	0.102	0.108	0.130	0.052
*C. soricinus*	1	882	0.034	0.017	0.034	—

Abbreviations: π, Nucleotide diversity; *F*_IS_, Inbreeding coefficient; *H*_E_, Expected heterozygosity; *H*_O_, Observed heterozygosity; *N*, Sample size; Private, Number of private alleles.

**FIGURE 6:**
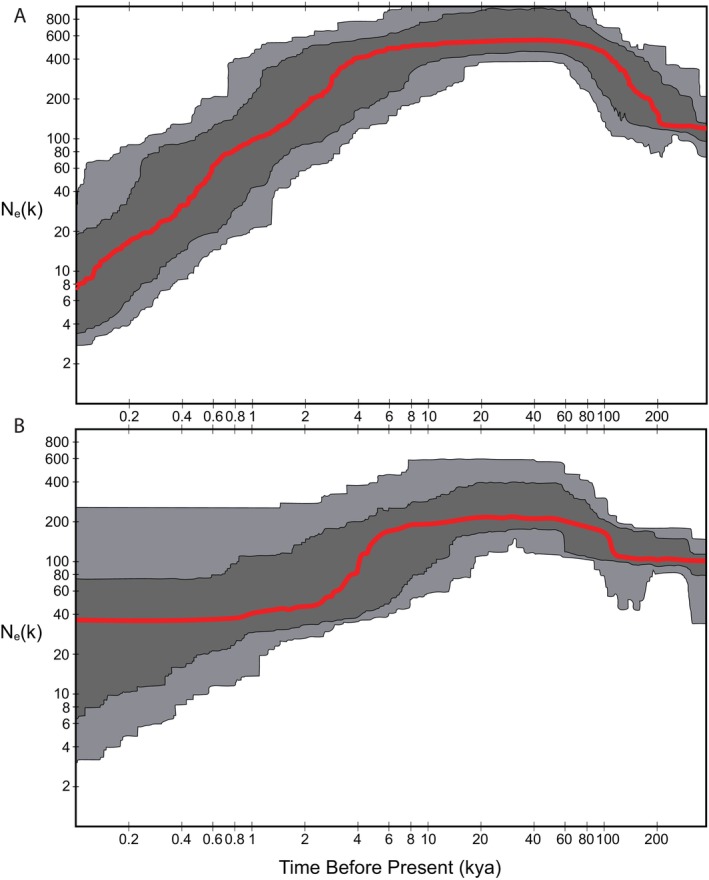
Stairway plots for estimated changes in effective population size (N_e_) for A: All *Cryptotis parvus parvus* west of the Mississippi River excluding samples from Chaves County, and B: All Chaves County individuals. Axes are shown as logarithmic. Red lines show change in median effective population size through time, dark shading is the 75% confidence interval and light shading is the 95% confidence interval on effective size estimates.

Stairway plots for demographic change for all *C. p. parvus* in the West group but excluding Chaves County samples showed relatively stable and large population sizes through the last glacial phase, followed by a steep decline in N_e_ coincident with a Holocene timeframe (Figure 6). For the Chaves County samples, again generally stable and robust values of N_e_ were estimated through the last glacial phase followed by a more modest decline through the Holocene (with broad confidence intervals).

### 3.5 Ecological Niche Modeling

**FIGURE 7:**
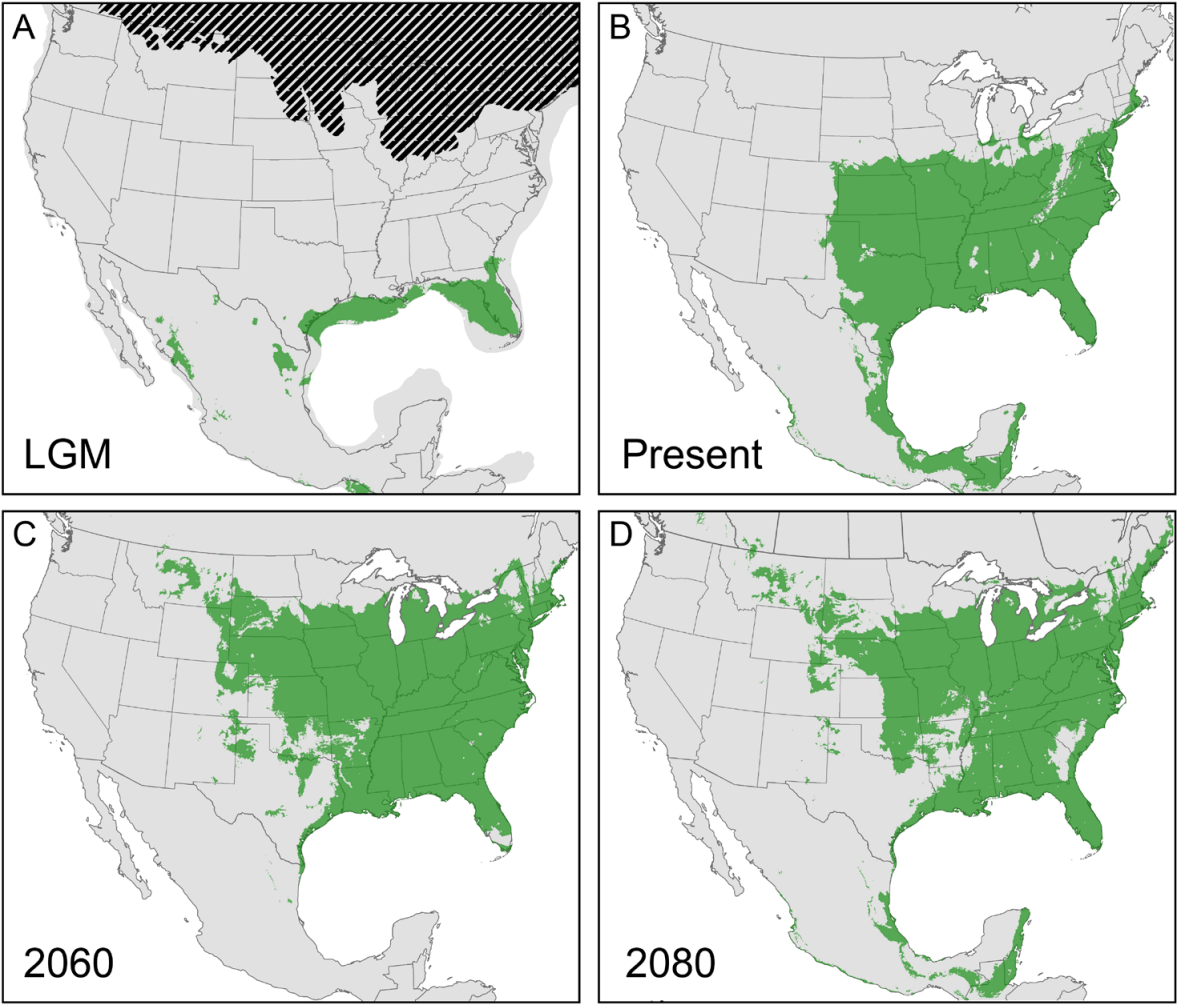
Ecological niche models for *Cryptotis parvus* (*sensu stricto*) showing projected niche using a logistic output format set to a robust threshold of suitability based on available locality data: (A) last glacial maximum; (B) present; (C) 2041–2060 future projection; (D) 2061–2080 future projection.

Niche models for *C. parvus* (sensu stricto) provide insight into understanding distributional change that may have strongly influenced evolutionary differentiation among lineages. The model for the present timeframe closely matches the observed distribution of *C. parvus* , based on sampling for models presuming a current distribution of this species that is restricted north of Mexico, including potential niche space in westernmost peripheral areas (Figure 7). In addition, the model does predict niche stretching south through Mesoamerica. However, there is no suitable niche space predicted in the region of Chaves County, New Mexico, the locality that currently supports a highly distinct lineage of shrews, suggesting that the persistence of *Cryptotis* shrews in these areas is due to factors other than a climatic envelope. The hindcast projection to the Last Glacial Maximum suggests a dramatic southern shift in distribution at this time, where much of the projected range is on the exposed continental shelf coincident with lowered sea levels. The projected distribution is also more fragmented into regions, including Florida, west of the Mississippi, and multiple regions within Mexico. Future‐cast projections strongly suggest potential for a continued northward distributional shift, with greater fragmentation of available niche in western regions, and decreased suitability of niche in southern regions. The exception to this is a corridor of suitable niche connecting the U.S. with Mesoamerica, recovered in models from all timeframes.

## 4 Discussion

We performed this study explicitly to investigate the evolutionary distinction and distribution of lineages among shrews of the *C. parvus* group through time, and to use this knowledge to further advance conservation of biodiversity more broadly, by highlighting benefits of our methods, and continued pitfalls for recognizing and managing significant conservation units. As such, the impetus for this work has been guided by the need for conservation decision‐making (Hohenlohe et al. 2021). Based on a combination of maternally inherited Cytb data and thousands of bi‐parentally inherited nuclear SNP loci, we have established that (1) the current taxonomy of least shrews does not accurately reflect the existing species diversity or systematic relationships among these taxa; (2) western peripheral populations that are currently listed as a single threatened taxon constitute two highly distinct lineages, one of which represents an endemic taxon within Chaves County, New Mexico. This Chaves County lineage is divergent across both neutral and non‐neutral loci reflecting extended allopatric differentiation from other populations as well as local adaptation, although both niche model projections and multi‐lineage sympatry in this region have negative implications for future integrity of this geographic isolate; (3) based on our genetic analyses, niche modeling, and comparative evidence from the literature, the *C. parvus* group is a model for broader biodiversity linkages between taxa currently distributed in eastern North America and taxa associated with Mesoamerica.

### 4.1 Revised Systematic Relationships and Taxonomic Updates

#### 4.1.1 Range‐Wide Considerations

Based on both mitochondrial and nuclear datasets, samples of the subspecies *C. p. floridanus* are unambiguously highly divergent from the remainder of the *C. parvus* group, and in fact are the sister taxon to all other species within the group, considering all available data. This includes multi‐locus data on both *C. berlandieri* and *C. soricinus* , as well as mitochondrial data for two additional species (*C. orophilus* and *C. tropicalis* ). Given all results, including no evidence of gene flow between *C. p. floridanus* and *C. p. parvus*, the former warrants recognition as an independent species, hereafter *Cryptotis floridanus*, Miller 1912. Given a paucity of samples from the southeast U.S. available for genomic analysis, further sampling of shrews from this region will be essential for investigating reproductive isolation, and the interactions between *C. floridanus* and *C. parvus* as environments continue to change. Hutchinson (2010) found strong evidence based on distinct differences in both morphology and genetics between *C. parvus* and *C. floridanus* and also supported a change of taxonomic status to recognize *C. floridanus* but found no evidence for additional taxa. For taxa of the *C. parvus* group distributed through Mexico and Mesoamerica, it is evident from our data that multiple species exist, and we cannot refute recognized taxonomic designations for species distributed through Mesoamerica, including *C. soricinus* , *C. tropicalis* , *C. orophilus*, and *C. berlandieri* (the latter with a distribution limited in the north to southern Texas). However, divergence between *C. tropicalis* and *C. orophilus* is relatively recent and based only on mitochondrial data. Further, it is possible that the specimens that we have to represent these species are simply being named based on their locality of collection in relation to the presumed species distribution and could conceivably be a different species of shrew. This highlights the continued lack of available samples for comparative phylogenetic and systematic appraisal for this entire region, where species richness among *Cryptotis* shrews is very likely higher than we currently recognize, even given our analyses.

There is substantially more diversity within *C. parvus* (sensu stricto), in addition to *C. floridanus* , than previously understood. He et al. (2015) recognized an evolutionary dichotomy across the Mississippi River that was subsequently suggested to represent two species, one in the west ( *C. parvus* ) and one in the east (*C. harlani*; Woodman 2018, 43), although based on only a few specimens. We have confirmed this divergence between west and east. In addition, the depth of mitochondrial divergence between these taxa is roughly equivalent to the divergence between *C. orophilus* and *C. tropicalis* . However, evolutionary depth, especially from a single locus, does not dictate the propensity for gene flow, and our admixture analysis clearly shows a low level of incomplete lineage sorting remains between these taxa for the loci we sampled. Further, the available specimens from the region spanning the Mississippi River are scant within digitized databases, and whereas this river may form a considerable barrier in the south, many species with phylogeographic divergence across the Mississippi River also exhibit substantial range overlap and gene flow in northern areas (e.g., Cassidy and Galbreath 2023). We therefore recognize these geographically discrete lineages as subspecies, hereafter *C. p. parvus* west of the Mississippi, and *C. p. harlani* east of the Mississippi. We also strongly recommend increased sampling of least shrews from mid‐western and eastern U.S. states on both sides of the Mississippi River to assess more fully the distinction between these taxa. Until this occurs, minimally, conservation and management decision‐making can be based on a reasonable re‐arrangement of named and geographically diagnosable subspecies (Dufresnes et al. 2023).

Within *C. p. parvus*, we recovered seven distinct genomic clusters of individuals which are also surprisingly consistent with geographic regionalization (Figures 1 and 2). Six of these clusters (1A–1F; Figure 1) are closely related but regionally discrete. Despite a signal of demographic change that supports recent range expansion when considering these six clusters collectively, it is possible that these populations constitute an example of in situ differentiation at a fine geographic scale that reflects local habitat refugia. For instance, a population associated with Bosque Redondo, De Baca County, along the Pecos River of New Mexico, and a population associated with Rockefeller Wildlife Refuge in southwestern Louisiana are both strongly supported based on all SNP loci and on the neutral SNP dataset (Figure 5, Figure S2), suggesting not only endemic diversity in these localities but potentially small populations having experienced strong drift. Further sampling from these areas and intervening space might clarify whether genetic distinction represents endemic diversity or simply fine‐scale isolation by distance. Finally, cluster 1G (Figures 1 and 2) constitutes a single population in Chaves County, New Mexico, that is distinct across all nuclear locus sets, with new implications for ongoing management in this region.

#### 4.1.2 Conservation Priorities for Western Peripheral Populations

Given the wildlife management priorities for *C. parvus* within New Mexico (NMDGF 2020), a primary finding of our study was that these western peripheral populations represent two distinct taxa. One is associated with Chaves County, and one is associated with the remaining geographic extent of *C. parvus* west of the Mississippi River. All but one individual from Chaves County were collected within wetlands associated with the unique and complex hydrology of the Roswell Artesian Basin (Land and Huff 2010). The single individual collected from outside of this wetland complex (FT644; within Cluster 1B of *C. p. parvus*; Table S2) was also genetically divergent from all shrews collected within the wetland areas (Figures 1 and 2). This individual was the furthest north sample within Chaves County and was collected from the periphery of the hydrologic area, suggesting that two shrew lineages occur in parapatry through this region. Wildlife managers should carefully monitor the region spanning the wetland complex, the remainder of the Pecos drainage basin, and the surrounding high plains for evidence of changing population demographics between western lineages. Genomic evidence suggests that the Chaves County lineage is demographically relatively stable, reflecting a buffered environment. Given that environmental perturbation (e.g., habitat disturbance or climate variability) is a leading cause of demographic change within small mammals as opposed to any influence of scientific collecting, modest specimen sampling through time should not substantially influence natural population trajectories (Hope et al. 2018). Continued development of specimen resources would provide critical insight into contemporary immigration of other lineages, potential gene flow, subsequent consequences for local adaptive fitness, and continued demographic trajectories across all populations.

The Chaves County lineage is well resolved as monophyletic based on nuclear data, yet was not diagnosable based solely on the Cytb data. This finding is counter to the understanding that mitochondrial genomes evolve on average four times faster than nuclear genomes (Allio et al. 2017). Further, Cytb data within this study exhibited deep pairwise divergence among other lineages. Such discordance between mitochondrial and nuclear data is increasingly common as genomic studies become more advanced (Coates et al. 2018). Primary explanations for such discordance include incomplete lineage sorting (e.g., Linck et al. 2019), hybridization (e.g., Colella et al. 2019), genetic drift, or selection in one or both sets of loci (e.g., da Fonseca et al. 2008), although the latter is normally considered to increase rates of mitochondrial evolution through directional selection.

Any of these dynamics may be occurring in least shrews. For instance, if a few ancestral mitochondrial haplotypes common among populations further east become fixed within the Chaves County population, for instance through a bottleneck, they may have persisted within this population in isolation, while the nuclear genome progressively diverged, resulting in unsorted (nonunique) mitochondrial DNA but divergent nuclear DNA. In terms of hybridization, mitochondrial capture among mammals has been documented in chipmunks (e.g., Good et al. 2008) and other mammals, where infrequent hybridization leaves a signal of the maternally inherited mitochondrial DNA from one lineage, while retaining the nuclear signature from the other lineage through a process of back‐cross breeding of the hybrids with the original lineage. This most often occurs through sporadic contact and interbreeding between genetic lineages that experience occasional geographic proximity.

Our data support two episodes of mitochondrial capture between least shrew lineages, once across the Mississippi River in Southern Louisiana and again in southern Texas. The latter transition zone in Texas is a recognized phylogeographic break for multiple other mammal species (Andersen and Light 2012; Herrera 2022). Given that Last Glacial environments in the southwestern U.S. and northern Mexico were considered to be more mesic, with cooler climates that facilitated pluvial lakes (e.g., Lake Palomas) and more extensive playa wetlands across the now relatively arid plains east of New Mexico, we suggest that historic (pre‐Holocene) connections between the Chaves County lineage and the remainder of *C. p. parvus* resulted in limited gene flow and subsequent mitochondrial capture of mitochondrial haplotypes from other populations of *C. p. parvus*. These haplotypes then propagated through the Chaves County lineage while maintaining a divergent nuclear signal (Andersen et al. 2021). A final possibility is that nuclear divergence of the Chaves County lineage has been rapid compared with the mitochondrial genome due either to drift (Wiens et al. 2022) or to directional selective pressure from the unique environments associated with the isolated habitats where this lineage is found (Bi et al. 2019). Given no evidence within the Chaves County lineage of a bottleneck and relatively high and stable effective population size through the Holocene (but with broad confidence limits; Figure 6), coupled with substantial divergence of this population across both neutral and non‐neutral loci (Figure 3), we suggest a combination of mitochondrial capture and local adaptations have contributed to uneven divergence between genomes.

Considering taxonomy and systematics, a lack of reciprocal monophyly between Chaves County and the remainder of *C. p. parvus* renders this lineage a subspecific unit. However, it is strongly supported as an evolutionarily significant unit based on all SNP loci, a demographically independent management unit based on neutral loci, and under endemic divergent selection as an adaptive unit based on non‐neutral loci (Funk et al. 2012). Hope and Frey (2022) found a similar evolutionary legacy among populations of least chipmunks, whereby a lack of reciprocal monophyly of an isolated population of conservation concern complicated the designation and recognition of taxonomic units. In this case, subspecies status was warranted based on diagnosability through multiple lines of evidence including both evolutionary and ecological criteria (Hope and Frey 2022). Despite Chaves County shrews lacking reciprocal monophyly with other lineages within *C. parvus* , they are unambiguously diagnostic based on nuclear data and biogeographic associations. We therefore recognize *Cryptotis* shrews associated with wetlands of the Roswell Artesian Basin as a diagnosable subspecies as described below.

We also strongly urge additional study of the ecological and morphological distinction of this taxon from others that might bolster the evidence from genetic data in support of this subspecies. For instance, Hafner and Shuster (1996) reported morphological distinction of Chaves County shrews from others through the western periphery based on two cranial measurements, instead aligning this taxon with *C. berlandieri* and with a southern origin. Our combined genetic analyses clearly show that Chaves County shrews are more closely related to other shrews within *C. parvus* , likely with an eastern origin, as seen for other species that may have experienced source‐sink dynamics associated with mesic refugia through their evolutionary history (Hafner 1993). Finally, additional genomic surveillance across peripheral populations should provide further perspective on taxonomic status. If the Chaves County population were found to be reproductively isolated from other populations, it would lend strong support for ecological speciation and recognition of a new species, despite a lack of reciprocal monophyly (Schluter 2009).

#### 4.1.3 *Cryptotis parvus neomexicanus n. subsp*.

**Etymology**—This subspecies is named for its endemic distribution within New Mexico, USA.

**Material Examined**—Holotype: Catalog number MSB:Mamm:271290, a male specimen collected by David J. Hafner on 27 September 1986. Skin, skull, postcranial skeleton, frozen kidney, and frozen liver are deposited in the Museum of Southwestern Biology (University of New Mexico, Albuquerque). Type locality: “4 mi. N, 7 mi. E Roswell”, at a ciénega wetland on Bitter Lake National Wildlife Refuge, Chaves County, New Mexico. Paratypes: In Chaves County, New Mexico, 12 additional specimens referred to this group as paratypes were collected from 4 areas of the Roswell Artesian Basin hydrologic system: eight from several locations at Bitter Lake National Wildlife Refuge (MSB:Mamm:271289, 329884, 329886, 329889, 329914, 329916, 329917); two from Bottomless Lakes State Park (MSB:Mamm:329902, 329903); two from the Bureau of Land Management Overflow Wetland (MSB:Mamm:329898, 329900); and one from Dexter National Fish Hatchery (MSB:Mamm:345442).

**Diagnosis**—Genomic evidence based on 22,786 single nucleotide polymorphisms identifies the holotype and paratypes as forming a monophyletic clade through Maximum Likelihood phylogenetic analysis, considering all loci, or when considering only neutral, or only non‐neutral loci. All 13 specimens were unambiguously assigned as an evolutionarily independent taxon based on the genomic admixture analysis (Figure 2), the clustering plots for all loci, neutral loci, and outlier loci (Figure 3), and the RAxML nuclear phylogeny (Figure 5). The holotype and a paratype (MSB:Mamm:271289), in addition to 13 other specimens from Bitter Lake National Wildlife Refuge (MSB:Mamm:271292, 271293, 271315, 272868, 272869, 272870, 272871, 272872, 272873, 272896, 272897, 272898, 272899) formed a cluster distinct from other examined western *C. p. parvus* based on 24 presumptive allozyme loci (Hafner and Shuster 1996). The Bitter Lake specimens shared different major alleles at six allozyme loci and had four unique alleles compared to western populations of *C. p. parvus* (Hafner and Shuster 1996). The cranium of *C. p. neomexicanus* differs from western populations of *C. p. parvus* in that larger individuals have a shorter palate relative to the postpalatal cranial length (Hafner and Shuster 1996). In external appearance, *C. p. neomexicanus* is overall darker than western populations of *C. p. parvus*. In *C. p. neomexicanus*, the dorsal pelage is dark gray and the ventral pelage and dorsal surface of feet are a slightly paler gray than the dorsum. In contrast, the pelage in western *C. p. parvus* is relatively pale with a brownish dorsum and whitish venter and dorsal surface of the feet.

**Remarks**—Means (mm) and ranges (in parentheses) for external measurements of 12 specimens from Chaves County are: Total length, 77.8 (71–88); tail length, 16.3 (14–20); hindfoot length, 10.9 (10–11.5); ear length, 4.2 (2.5–5.7); mass, 4.2 g (2.5–5.7 g). Means (mm) and ranges (in parenthesis) for cranial measurements of 5 specimens from Chaves County are: Condylobasilar length, 15.81 (15.45–16.32); cranial breadth, 7.93 (7.68–8.35); interorbital breadth, 3.66 (3.46–3.84); palatal length, 6.36 (6.24–6.72); length of upper toothrow, 5.61 (5.38–5.81); length of mandible, 6.28 (6.08–6.47). Distribution: The range of *C. p. neomexicanus* is likely limited to the Roswell Artesian Basin, a unique wetland system that includes Bitter Lake National Wildlife Refuge and Bottomless Lakes State Park, which are recognized as internationally important wetlands (https://rsis.ramsar.org/ris/1917). The Roswell Artesian Basin is located in Chaves and Eddy counties, New Mexico, and extends from the Pecos River in the east, westward to near the Lincoln County border, and from Macho Draw in northern Chaves County southward to the Seven Rivers Hills near Carlsbad in central Eddy County (https://geoinfo.nmt.edu/resources/water/projects/bwa/roswell/home.html). Specimen records definitively referred to this subspecies based on genomic data are from four general locations in Chaves County: Bitter Lake National Wildlife Refuge, Bottomless Lakes State Park, Bureau of Land Management Overflow Wetland, and Dexter National Fish Hatchery. A specimen of *C. parvus* collected 10 June 1961 from 10.35 miles SSE of Artesia, Eddy County, 3286 ft. elevation (University of Colorado Museum catalog number 10974) is referrable to this subspecies on a geographic basis. Subsequent surveys in Eddy County have failed to document the shrew (NMDGF 2020).

### 4.2 Comparative Evolution of Eastern North America and Mesoamerica

A growing number of studies have reported evolutionary linkages between flora and fauna distributed through temperate forests of eastern North America and forested regions of Mexico and Mesoamerica, summarized through references therein by Martin and Harrell (1957) for both fauna and flora and by Stull (2023) more extensively for floral species. Further, these authors offered support for a mechanism that drives both continued, or at least intermittent, connections between these regions, and for the processes of evolution in response to climatic cycling of the Pleistocene that drive distributional and demographic change. This results in the generation of discrete lineages associated with modern distributions. We suggest that the *C. parvus* group of shrews provides a further clear example of this dynamic. Recent work has also developed evidence for similar histories of diversification across other small mammals including *Glaucomys volans* (Kerhoulas and Arbogast 2010), *Peromyscus leucopus* , the *Neotoma floridana* group, and *Sigmodon hispidus* (Herrera 2022). Small mammals may provide important insight as they are less vagile than birds that also show many instances of similar histories but often with more “noise” due to long‐distance migration and rapid movement (e.g., Smith et al. 2011). In addition, small mammals generally evolve and move more quickly than plant taxa, and have greater environmental tolerances than amphibians and reptiles allowing them to span ranges through both the eastern U.S. and across Mesoamerica more easily.

Like *C. parvus* , many taxa with similar distributions have evolutionarily distinct lineages associated with Florida (and/or more broadly within the eastern U.S.), with west of the Mississippi, east of the Mississippi, and with potentially multiple lineages associated with regional sierras through Mesoamerica. Further, the relative ages of these lineages are highly variable, strongly supporting a repeated cyclic process through deep time. Within the *C. parvus* group, both interspecific and infraspecific evolutionary divergences conform to this model of diversification through time. Our niche model predictions provide additional support for diversification in response to climate change (Figure 7). In particular, the Last Glacial Maximum projection highlights multiple isolated areas of potential niche that support a hypothesis of regional allopatry at this time. Coupled with a signal of elevated effective population sizes among lineages during the last glacial phase (Figure 6), the repeated southern shunt in distributions was accompanied by favorable ecological conditions at lower latitude, particularly through western and southern regions compared to the present timeframe. Niche model predictions across all time frames also showed available niche space along the corridor between the southern U.S. and Mexico, consistent with the coastal plain habitats of east Texas. This most likely constitutes the long‐term corridor for north–south shifts of populations between the eastern U.S. and Mesoamerica and is a critical region for maintaining suitable mesic habitats through glacial phases, as shown for amphibians (Barrow et al. 2017). Further, our niche model predictions were likely conservative in their extent based on strict filtering of occurrence data, as well as an observation that the current predicted niche does not capture all observed western and northern peripheral populations. An alternative interpretation is that the realized distribution of *C. parvus* beyond the predicted climatic niche is enabled by locally available habitats that persist outside of suitable climate parameters.

Following glacial phase spillover into Mesoamerica, and across the sierra complexes of Mexico, subsequent evolution in allopatry would ensue. Our future niche predictions give an impression of declining niche suitability through southwestern regions and Mesoamerica into the near future, and as climate warms and dries, suitable habitat likely will continue to retreat to higher elevation refugia in these areas. Coupled with anthropogenic loss of habitat in the present Holocene interglacial through multiple mechanisms, populations that may have persisted in small refugia across this region through previous interglacials may be at increased risk of modern extirpation or extinction (Kerhoulas and Arbogast 2010). Finally, a loss of available climatic niche space for shrews and many other taxa is of obvious conservation concern, where in these situations, populations must instead rely on biotic variables to persist, particularly including isolated relictual habitats that rely on other means than variation in temperature and precipitation to remain intact. The hydrologic wetlands associated with Chaves County, New Mexico, have seemingly supported *Cryptotis* shrews through the last glacial phase and the current interglacial. Considering this area has not been predicted as suitable climatic niche through any of the timeframes we modeled, the greatest threat to this population is therefore likely to be a loss or perturbation of this ground‐fed wetland. Similarly, maintenance of local mesic habitats throughout the distribution of these shrews is a critical consideration for conserving range‐wide diversity.

## 5 Conclusions and Future Priorities

Our combined genomic and phylogeographic analyses have allowed us to address conservation issues for *Cryptotis* shrews spanning multiple scales of perspective. This includes identifying high‐risk populations and lineages, refining our knowledge of nomenclature, species limits, and functional units on which conservation and management actions can be focused, and resolving repeated processes of evolution spatially, temporally, and across disparate taxonomic groups. However, implementing effective conservation still faces challenges. Predictions of continued range changes of populations of *Cryptotis* across boundaries between lineages will promote potential for gene flow through admixture and subsequent genomic consequences of hybridization. This has the potential to dilute, erase, or recombine novel diversity, a risk especially for locally adapted genotypes (Smith et al. 2011). Fortunately, advances in genomic methods for assessing the adaptive consequences of gene flow, and identifying particular genes of importance for maintaining populations have been rapid, and continue (Hohenlohe et al. 2021; Theissinger et al. 2023). In addition, our data demonstrate multiple instances of gene flow through the diversification of these shrews that likely will continue across zones of contact between taxa. Although hybrids are still not offered the same strength of protection through legislation, there is much progress in this regard (e.g., Quilodrán et al. 2020; Hirashiki et al. 2021; Heuertz et al. 2023). A leading option for promoting the evolutionary potential of shrews of the *C. parvus* group and biodiversity at large is to maintain intact and robust populations from across the entire distribution of these taxa. For shrews, this will require maintaining a multi‐tiered approach to refine our knowledge of the remaining species within the *C. parvus* group that were under‐sampled in this study. It will also require a greater understanding of contemporary genomic changes across populations by adopting novel molecular methods, particularly focused on functional perspectives. In addition, a greater understanding of the linkages between these shrews and their respective communities, from ecological, co‐evolutionary, and parallel evolutionary perspectives will be important. None of these things will be adequately realized without extensive future sampling of *Cryptotis* shrews (and other species) throughout North America (Colella et al. 2021). Molecular technology is improving to the point where genomic data from old museum specimens is becoming a game changer for understanding the genomic legacies of past biodiversity (Card et al. 2021). Understanding continued trajectories of change in biodiversity will strongly benefit from coordinated future specimen sampling, preservation, and global digitized access to these resources by the broader scientific and wildlife legislation community (Miller et al. 2020).

## Author Contributions

**Tommy M. Galfano:** conceptualization (equal), data curation (equal), formal analysis (lead), writing – original draft (lead), writing – review and editing (supporting). **Tommy M. Herrera:** conceptualization (equal), data curation (equal), formal analysis (supporting), writing – original draft (supporting), writing – review and editing (equal). **John B. Bulger:** conceptualization (equal), data curation (equal), writing – original draft (supporting), writing – review and editing (supporting). **James N. Stuart:** conceptualization (equal), data curation (equal), writing – original draft (supporting), writing – review and editing (supporting). **Jennifer K. Frey:** conceptualization (equal), data curation (equal), writing – original draft (supporting), writing – review and editing (supporting). **Andrew G. Hope:** conceptualization (equal), data curation (equal), formal analysis (supporting), funding acquisition (lead), writing – original draft (lead), writing – review and editing (lead).

## Conflicts of Interest

The authors declare no conflicts of interest.

## Supporting information


**Table S1.** Phylogenomic analysis of wide‐ranging least shrews refines conservation priorities and supports a paradigm for evolution of biota spanning eastern North America and Mesoamerica.
**Table S2.** Phylogenomic analysis of wide‐ranging least shrews refines conservation priorities and supports a paradigm for evolution of biota spanning eastern North America and Mesoamerica.
**Table S3.** Institutional Museum Codes.

**Figure S1.** All admixture plots showing distinct genomic clusters of individuals of *Cryptotis* based on *K* = 2–12, and assignment of SNP loci to each cluster. Sample labels correspond to specimen data as appended within the figure and reflected in Table S2. Also provided is the *K*‐Estimator Chart showing *K* = 11 as the best value.

**Figure S2.** Bayesian mtDNA cytochrome b chronogram as estimated in BEAST for all putative samples of *Cryptotis parvus* (sensu stricto), excluding *C. p. floridanus* and outgroups. Bars next to terminal branches are colored according to cluster assignment based on nuclear SNP loci as reported in Figure 2. Samples without bars were not sequenced for nuclear data. Black dots show ≥ 0.95 posterior probability estimates at the proceeding node. The scale bar shows tree depth in millions of years before present based on an estimated mutation rate of 5.5% per million years. The two primary reciprocally monophyletic clades recovered represent samples collected from either west or east of the Mississippi River.

**Figure S3.** RAxML phylogeny for all samples with available nuclear data, based only on putatively neutral loci (21,451 SNPs). Black dots show ≥ 0.95 bootstrap support at the proceeding node. Bars next to terminal branches are colored according to cluster assignment based on nuclear SNP loci as reported in Figure 2.

**Figure S4.** RAxML phylogeny for all samples with available nuclear data, based only on outlier loci (1335 SNPs). Black dots indicate ≥ 0.95 bootstrap support at the proceeding node. Bars next to terminal branches are colored according to cluster assignment based on nuclear SNP loci as reported in Figure 2.

## Data Availability

Data for all specimens examined is provided in Table S2. All CytB sequences have been deposited to GenBank (Accessions PQ278313—PQ278406). All ddRAD data are available via FigShare (https://doi.org/10.6084/m9.figshare.26970568.v2).
