# Supplementary material for: Phylogenomic Analysis of Wide‐Ranging Least Shrews Refines Conservation Priorities and Supports a Paradigm for Evolution of Biota Spanning Eastern North America and Mesoamerica
Source: Ecol Evol. 2025 May 12;15(5):e71263. doi: 10.1002/ece3.71263 (PMC12066985; doi:10.1002/ece3.71263)

Sample	Label
1	COCRWDMNS16603
2	FLCFLUF31660
3	FLSCFLUF33466
4	FLSFLUF31133
5	KSCRWNK306540
6	KSERWMSB305705
7	KSERWNK296152
8	KSEUNMSB305706
9	KSSWRWNK306133
10	KSSWRWNK306166
11	KSSWRWNK306167
12	LACNMSLSU29129
13	LACUNLSU26721
14	LAENMSLSU25419
15	LAENMSLSU29128
16	LAEUNLSU28649
17	LAEUNLSU28650
18	LAWNMSLSU28942
19	LAWUNLSU28941
20	LAWUNLSU29015
21	LAWUNLSU34215
22	LAWUNLSU34218
23	MXTXSLSU34413
24	NMNRWFT647
25	NMNRWMSB271323
26	NMNRWMSB271324
27	NMNRWMSB271655
28	NMNRWNK305195
29	NMNRWNK305196
30	NMNRWNK305197
31	NMNRWNK305198
32	NMNRWNK305199
33	NMNRWNK305200
34	NMNRWNK305202
35	NMNRWNK305206
36	NMNRWNK305209
37	NMNRWNK305210
38	NMNRWNK305211
39	NMNUNFT653
40	NMNUNNK306952
41	NMNUNNK306954
42	NMNUNNK306959
43	NMSNMSET203
44	NMSNMSET460
45	NMSNMSET465
46	NMSNMSFT642
47	NMSNMSFT645
48	NMSNMSFT654
49	NMSNMSMSB271289
50	NMSNMSMSB271290
51	NMSNMSNK305208
52	NMSRWFT644
53	NMSUNFT656
54	NMSUNFT658
55	NMSUNFT659
56	TXCNMSAS11116
57	TXCNMSAS12494
58	TXCRWAS8191
59	TXCRWAS8192
60	TXENMSTAM63771
61	TXNRWAS14356
62	TXSENMSMSB196185
63	TXSTXSAS13679
64	TXSTXSNK305214
65	VAVAMSB310263

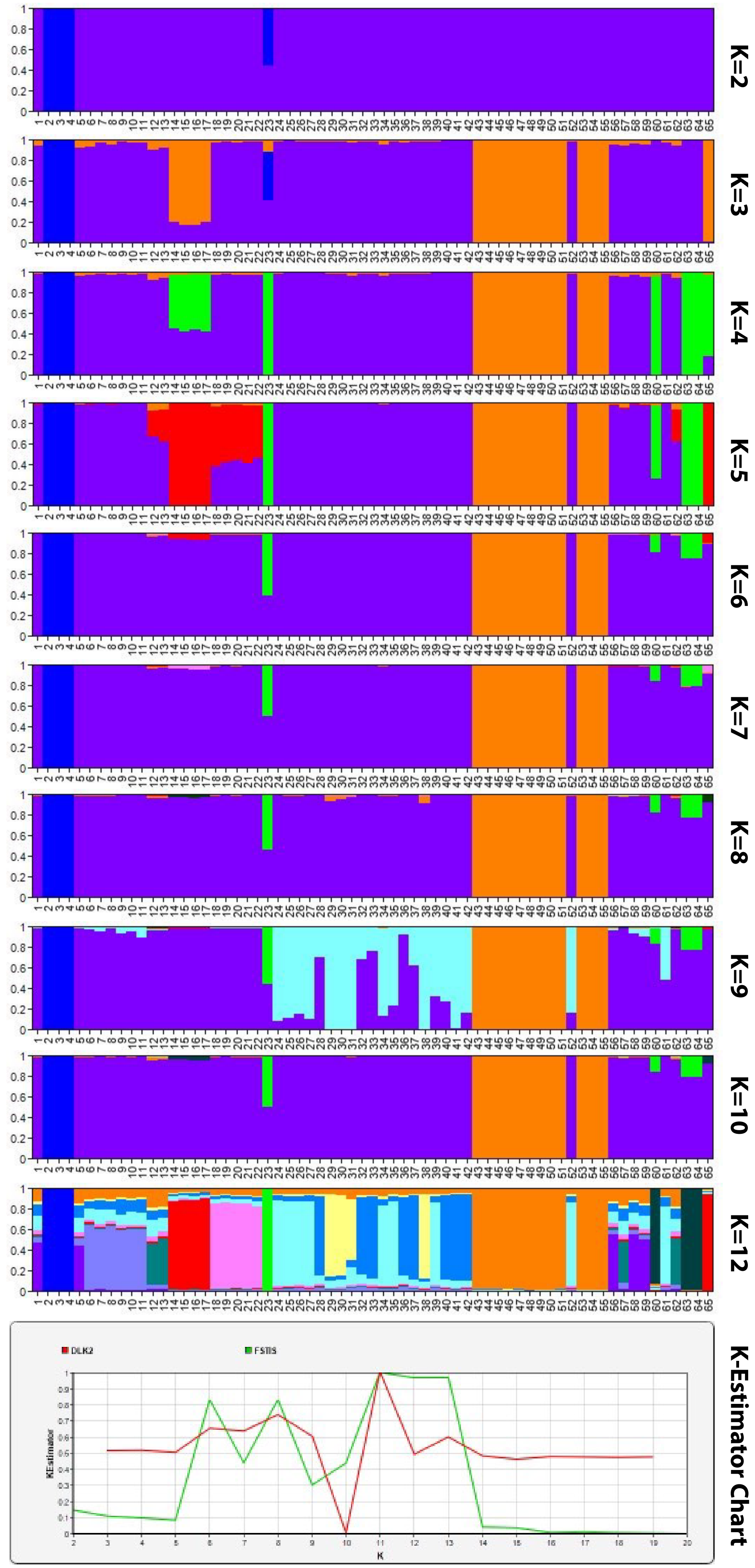

Supplement: Supplementary file 2 — Figure S1. All admixture plots showing distinct genomic clusters of individuals of Cryptotis based on K = 2–12, and assignment of SNP loci to each cluster. Sample labels correspond to specimen data as appended within the figure and reflected in Table S2. Also provided is the K‐Estimator Chart showing K = 11 as the best value. [file ECE3-15-e71263-s005.pdf]
