# Supplementary figures and images for: Phylogenomic Analysis of Wide‐Ranging Least Shrews Refines Conservation Priorities and Supports a Paradigm for Evolution of Biota Spanning Eastern North America and Mesoamerica
Source: Ecol Evol. 2025 May 12;15(5):e71263. doi: 10.1002/ece3.71263 (PMC12066985; doi:10.1002/ece3.71263)

Appendix Figure A1.

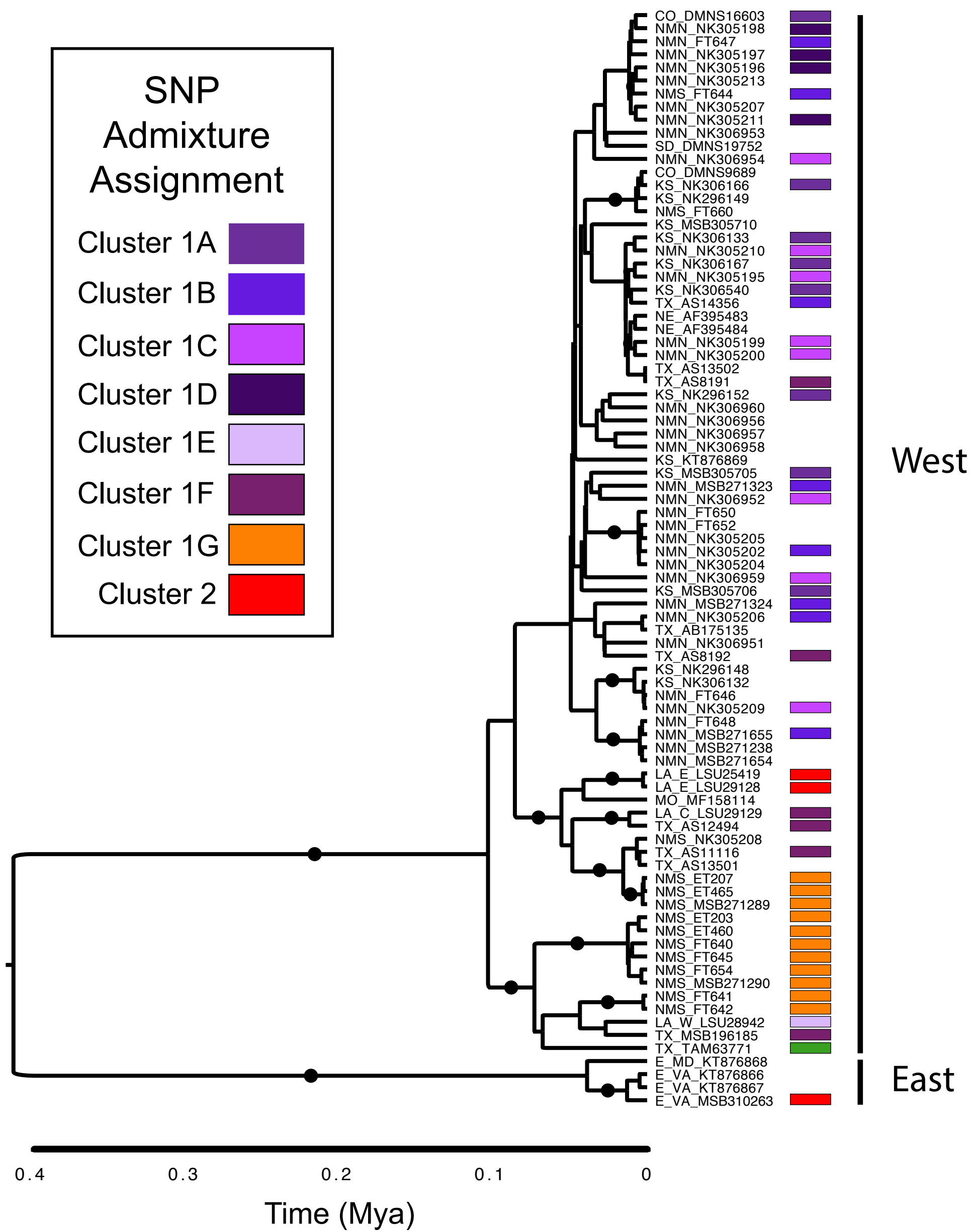

Supplement: Supplementary file 3 — Figure S2. Bayesian mtDNA cytochrome b chronogram as estimated in BEAST for all putative samples of Cryptotis parvus (sensu stricto), excluding C. p. floridanus and outgroups. Bars next to terminal branches are colored according to cluster assignment based on nuclear SNP loci as reported in Figure 2. Samples without bars were not sequenced for nuclear data. Black dots show ≥ 0.95 posterior probability estimates at the proceeding node. The scale bar shows tree depth in millions of years before present based on an estimated mutation rate of 5.5% per million years. The two primary reciprocally monophyletic clades recovered represent samples collected from either west or east of the Mississippi River. [file ECE3-15-e71263-s002.pdf]

Appendix Figure A2.

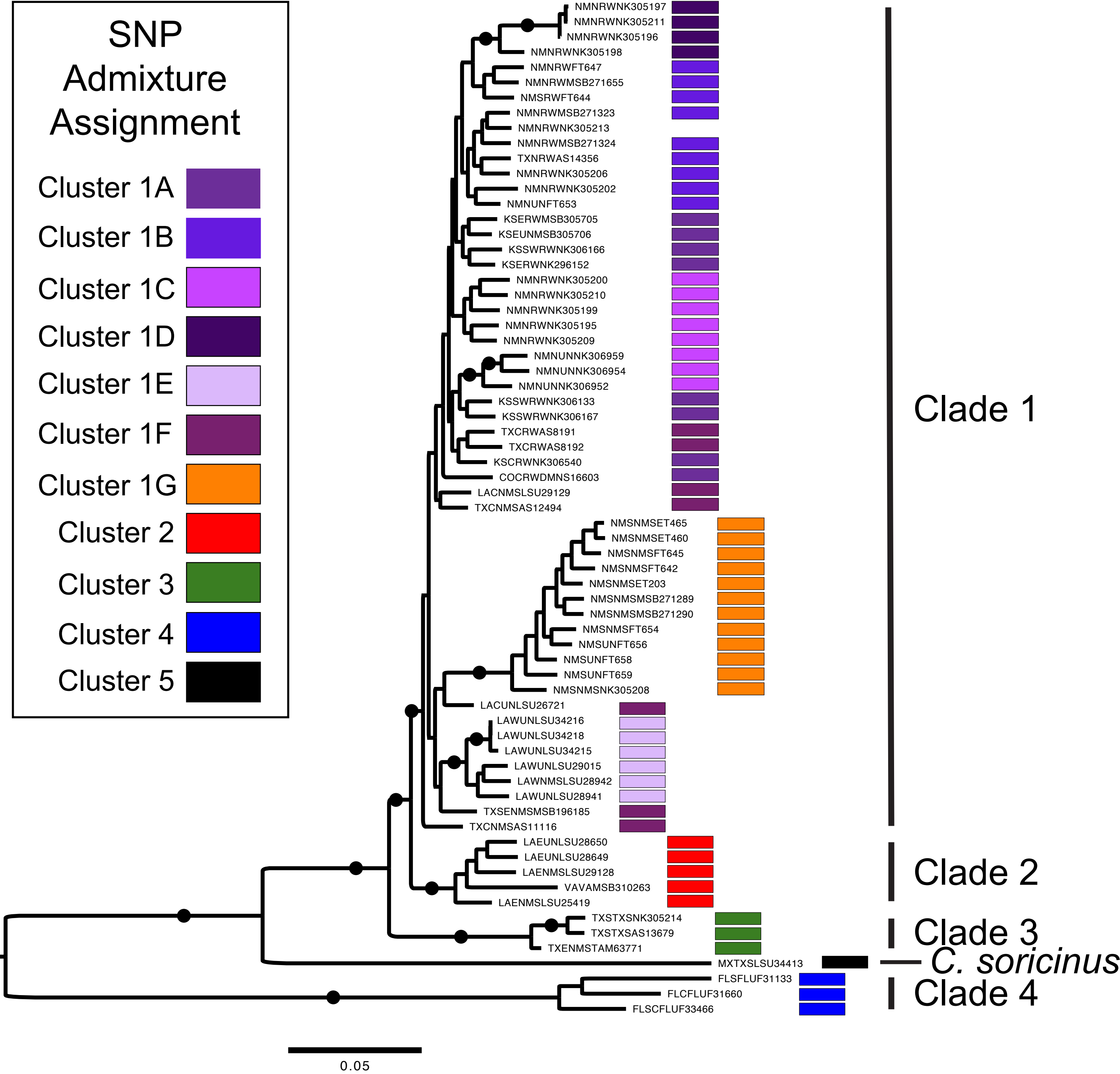

Supplement: Supplementary file 4 — Figure S3. RAxML phylogeny for all samples with available nuclear data, based only on putatively neutral loci (21,451 SNPs). Black dots show ≥ 0.95 bootstrap support at the proceeding node. Bars next to terminal branches are colored according to cluster assignment based on nuclear SNP loci as reported in Figure 2. [file ECE3-15-e71263-s003.pdf]

Appendix Figure A3.

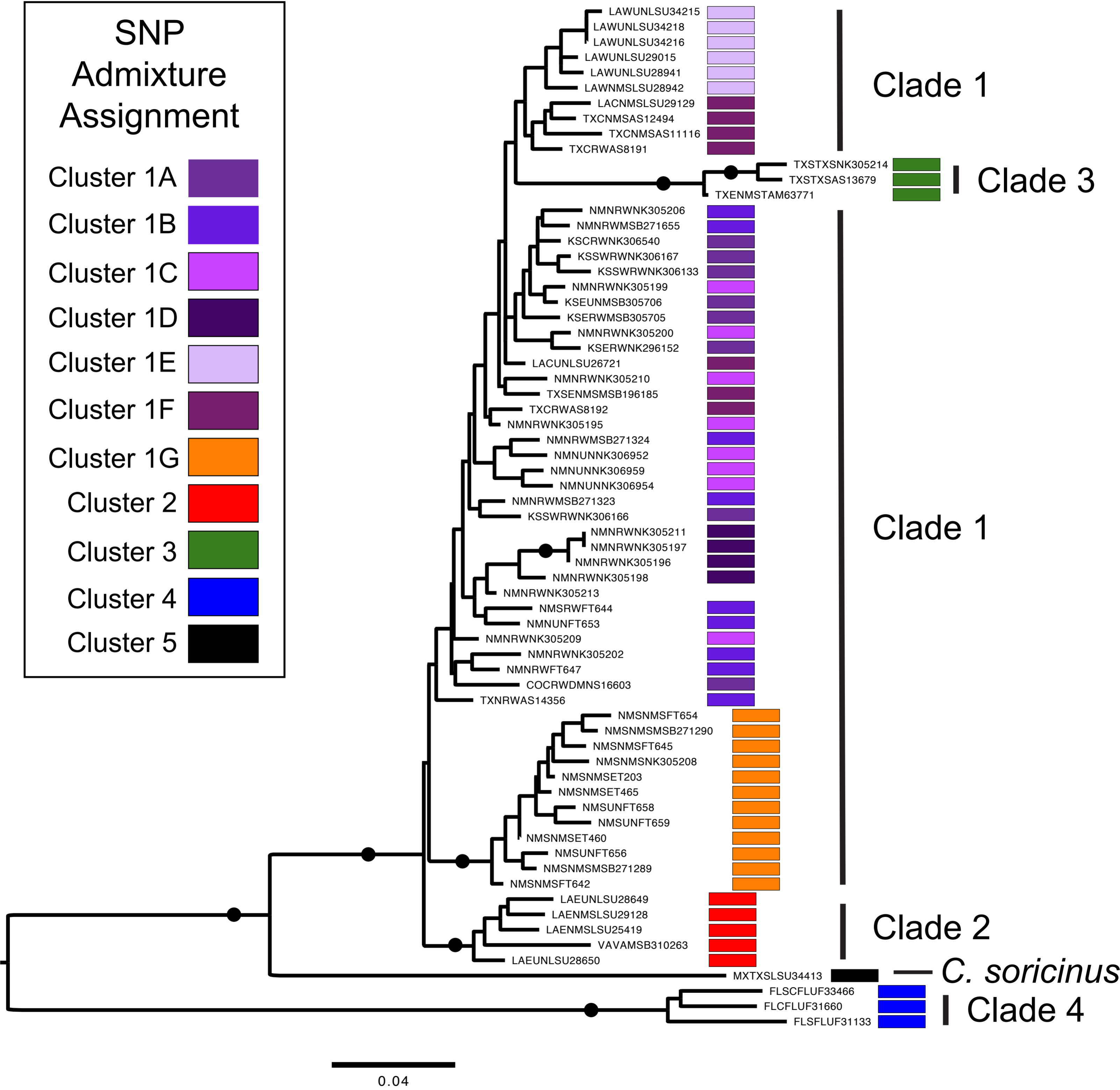

Supplement: Supplementary file 5 — Figure S4. RAxML phylogeny for all samples with available nuclear data, based only on outlier loci (1335 SNPs). Black dots indicate ≥ 0.95 bootstrap support at the proceeding node. Bars next to terminal branches are colored according to cluster assignment based on nuclear SNP loci as reported in Figure 2. [file ECE3-15-e71263-s004.pdf]
